# Dynamic encounters with red blood cells trigger splenic marginal zone B cell retention and function

**DOI:** 10.1038/s41590-023-01690-z

**Published:** 2023-12-04

**Authors:** Dan Liu, Benjamin Y. Winer, Marissa Y. Chou, Hanson Tam, Ying Xu, Jinping An, James M. Gardner, Jason G. Cyster

**Affiliations:** 1 Howard Hughes Medical Institute and Department of Microbiology and Immunology, University of California, San Francisco, San Francisco, CA USA; 2 Westlake Laboratory of Life Sciences and Biomedicine, Westlake University School of Life Sciences, Institute of Basic Medical Sciences and Westlake Institute for Advanced Study, Hangzhou, China; 3 Immunology Program, Memorial Sloan Kettering Cancer Center, New York, NY USA; 4 Diabetes Center and Department of Surgery, University of California, San Francisco, San Francisco, CA USA

**Keywords:** Marginal zone B cells, Spleen, Imaging the immune system

## Abstract

Spleen marginal zone (MZ) B cells are important for antibody responses against blood-borne antigens. The signals they use to detect exposure to blood are not well defined. Here, using intravital two-photon microscopy in mice, we observe transient contacts between MZ B cells and red blood cells that are in flow. We show that MZ B cells use adhesion G-protein-coupled receptor ADGRE5 (CD97) for retention in the spleen. CD97 function in MZ B cells depends on its ability to undergo autoproteolytic cleavage and signaling via Gα_13_ and ARHGEF1. Red blood cell expression of the CD97 ligand CD55 is required for MZ B cell homeostasis. Applying a pulling force on CD97-transfected cells using an optical C-trap and CD55^+^ beads leads to accumulation of active RhoA and membrane retraction. Finally, we show that CD97 deficiency leads to a reduced T cell-independent IgM response. Thus, our studies provide evidence that MZ B cells use mechanosensing to position in a manner that enhances antibody responses against blood-borne antigens.

## Abstract (web summary)

Cyster and colleagues show that CD97–CD55 interactions, which trigger Gα_13_–ARHGEF–Rho cytoskeletal signaling, are needed for proper MZ B cell positioning/retention in the spleen and for optimal antibody responses to T cell-independent antigens.

## Main

The spleen, the largest secondary lymphoid organ, is unique in having an open blood circulation. Central arterioles branch into open-ended terminal arterioles that release blood into the marginal sinus and red pulp^1–3^. Lymphoid regions sheathe the central arterioles and are termed white pulp cords. The interface between the white pulp and the red pulp, known as the marginal zone (MZ), contains a specialized population of MZ B cells as well as macrophages, dendritic cells and stromal cells^1–3^. Blood percolates from the marginal sinus through the MZ before reaching the red pulp and returning to circulation via the venous sinuses. As a result, MZ cells are rapidly exposed to blood-borne antigens. MZ cells are also thought to be extensively exposed to blood-borne cells, but the dynamics of these interactions have not been determined. MZ B cells have a poised, semiactivated state compared to follicular B cells, and they differentiate rapidly into plasma cells following antigen encounter. This property helps provide an early wave of antibodies targeted to blood-borne pathogens^4,5^.

Multiple cues act to position MZ B cells. The cells are attracted to the MZ by sphingosine-1-phosphate receptor-1, which responds to S1P that is abundant in blood^6^. Although known for their occupancy of the MZ, MZ B cells are motile and continually shuttle between the MZ and follicle^7,8^. This shuttling contributes to the delivery of blood-borne antigens to follicular dendritic cells for display to follicular B cells. Retention of MZ B cells within the MZ depends on integrin-mediated adhesion, and blockade of integrin function leads to their loss into blood circulation^8,9^. MZ B cell homeostasis also depends on the signaling molecules CNR2, PYK2, BTK, TAOK3 and NOTCH^2,10,11^. Despite this advanced understanding of MZ B cell positioning and homeostasis requirements, the mechanisms that MZ B cells use to sense their location with respect to blood flow are incompletely understood.

Adhesion G-protein-coupled receptors (GPCRs) are a subfamily of GPCRs typified by a long extracellular N-terminal fragment (NTF) and a C-terminal fragment (CTF) that corresponds to the GPCR domain^12,13^. CD97, or adhesion GPCR E5 (ADGRE5), is a member of the epidermal growth factor (EGF) repeat-containing subgroup of adhesion GPCRs that is widely expressed on hematopoietic cells^12^. Like most adhesion GPCRs, CD97 undergoes autoproteolytic cleavage at the GPCR proteolysis site (GPS) within the GPCR-activation-inducing domain. The NTF and CTF remain noncovalently bound via the GPCR-activation-inducing domain. A theme that has emerged from in vitro studies of several adhesion GPCRs is that their activation can be promoted by force being exerted on the NTF to expose a tethered ligand that is present at the N terminus of the CTF, enabling activation of the receptor^13,14^. The best-defined ligand for the CD97 NTF is the glycosylphosphatidylinositol-anchored membrane protein CD55 (refs. ^12,15^). Encounters between CD55^+^ cells and CD97^+^ cells under shear stress conditions can lead to extraction of the CD97 NTF^16,17^. CD97 and CD55 deficiency are associated with mild granulocytosis and reductions in spleen type 2 conventional dendritic cells^17–20^. However, the cellular changes caused by CD55 engagement of CD97 are not well defined. Moreover, the function of CD97 in other immune cell types is not understood.

Here, using intravital two-photon microscopy of the spleen, we observed interactions between MZ B cells and red blood cells (RBCs) in flow. We found that MZ B cells express high levels of CD97 and depend on this receptor and downstream signaling proteins Gα_13_ and ARHGEF1 for their retention and homeostasis. CD97 function was dependent on the expression of CD55 on RBCs, and the NTF of CD97 on both mouse and human MZ B cells was extracted by engagement with CD55^+^ RBCs under shear stress conditions. Using CD97-transfected HEK293T cells, optical trap measurements showed that pulling forces exerted on CD97 via CD55^+^ particles caused RhoA activation and cell membrane retraction. CD97 pathway-deficient mice mounted reduced T cell-independent IgM responses against a polysaccharide antigen. These findings support a model where MZ B cell mechanosensation of passing RBCs via CD97–CD55 interaction causes membrane retraction and thus cell retention and function within the spleen.

## Results

### Intravital imaging reveals MZ B cell–RBC contacts

**Fig. 1:**
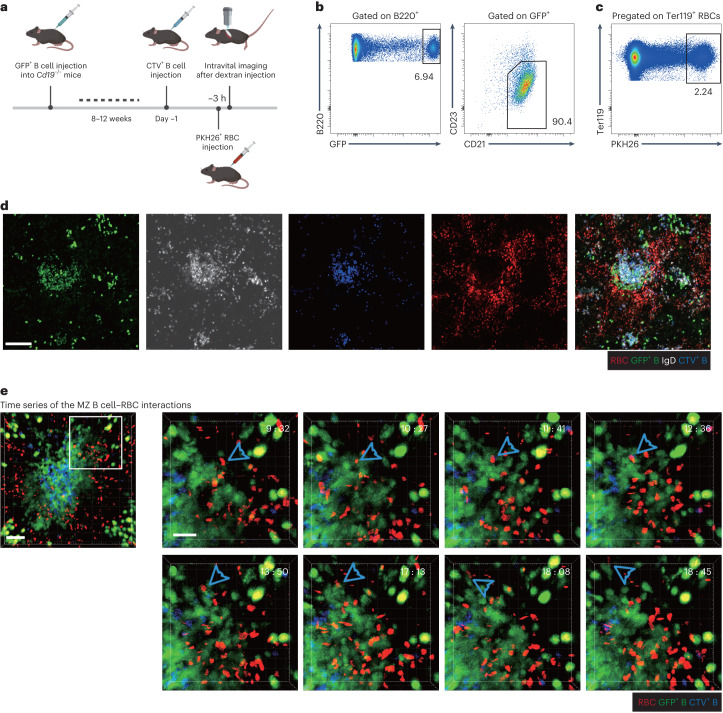
Intravital imaging reveals transient contacts between MZ B cells and RBCs. The behaviors of MZ B cells were observed with intravital two-photon microscopy in *Cd19*^−/−^ mice reconstituted with Ub-GFP^+^ B cells. CTV-labeled naive B cells were used to identify follicles. PKH26-labeled RBCs were intravenously injected 3 h before imaging, and 70-kD Texas Red dextran was introduced 30 min before imaging. **a**, Schematic diagram of the intravital imaging protocol. **b**, Representative flow cytometry profiles of MZ B cells among GFP^+^ B cells 8–12 weeks after reconstitution. **c**, A representative flow cytometry profile of PKH26-labeled RBCs in total splenic RBCs. **d**, Representative distribution patterns of PKH26-labeled RBCs (red), GFP^+^ B cells (green), CTV-labeled B cells (blue) and IgD^+^ B cells (white) in the spleen; scale bar, 100 μm. The section shown is representative of multiple cross-sections from at least three mice. **e**, Examples of contacts between RBCs and MZ B cells (blue arrowhead) in spleens. The region (120 μm × 120 μm) indicated with white box (left; scale bar, 40 μm) was zoomed in for the time series (right; scale bar, 20 μm). One RBC was tracked interacting with several different MZ B cells. The interaction starts at 9:32 and ends at 18:45. Time is indicated in minutes:seconds. See corresponding Supplementary Videos 2–4. Data are representative of five independent experiments.

MZ B cells are distinguished from follicular B cells by high expression of CD21 and IgM and low expression of CD23 and IgD^4,5^. To enable intravital two-photon microscopy of MZ B cells, we used transferred B cells from ubiquitin-green fluorescent protein (Ub-GFP)-expressing mice to *Cd19*-knockout (KO) mice that lack an endogenous MZ B cell population (Fig. 1a)^8^. After 8–12 weeks, the MZ was occupied by the transferred B cells, and the majority of the transferred GFP^+^ B cells had a CD21^hi^CD23^lo^ MZ B cell phenotype (Fig. 1b). Three hours before analysis, the mice were transfused with PKH26 (red) dye-labeled RBCs such that about 2% of RBCs in the recipient mice were labeled (Fig. 1c). The mice were also given transfers of CellTrace Violet (CTV)-labeled follicular B cells 1 or 2 d before to help identify lymphoid follicles. To guide expectations for the intravital imaging analysis, which can detect fluorescent cells at depths of up to 200 µm, thin sections were taken within 200 µm of the capsule and examined for the distribution of the transferred B cells and RBCs and for total IgD^+^ follicular B cells. This analysis showed that occasional small follicular structures could be detected at this depth, and these had GFP^+^ B cells superficially associated that were IgD^lo^ and thus were most likely MZ B cells (Fig. 1d). Clusters of large GFP-bright cells were also detected in the red pulp, distant from the B cell follicles, likely corresponding to plasma cells.

Scanning different regions of the spleen using intravital two-photon microscopy allowed for the identification of occasional areas with GFP^+^ MZ B cells near clusters of CTV^+^ follicular B cells (Fig. 1e and Supplementary Video 1). Labeled RBCs were observed passing near MZ B cells, and transient contacts between MZ B cells and RBCs could be detected (Fig. 1e and Supplementary Videos 2–4). Some RBCs encountered different MZ B cells during their movement (Fig. 1e and Supplementary Videos 2 and 3), and some MZ B cells were observed interacting with several RBCs (Supplementary Videos 2 and 4). In some regions of the MZ, the RBCs appeared to be moving in an irregular manner, perhaps within a turbulent and semiconfined space, whereas in other regions, the RBCs passed through the MZ with uniform flow (Supplementary Videos 2–4). Although we could readily detect RBC–MZ B cell contacts in each 20- to 30-min video, it is important to appreciate that only ~2% of the RBCs were labeled, and thus the contact frequency is expected to be 50-fold greater than imaged. These data establish that MZ B cells regularly contact RBCs in regions of flow within the MZ.

### CD97 is required for MZ B cell homeostasis

**Fig. 2:**
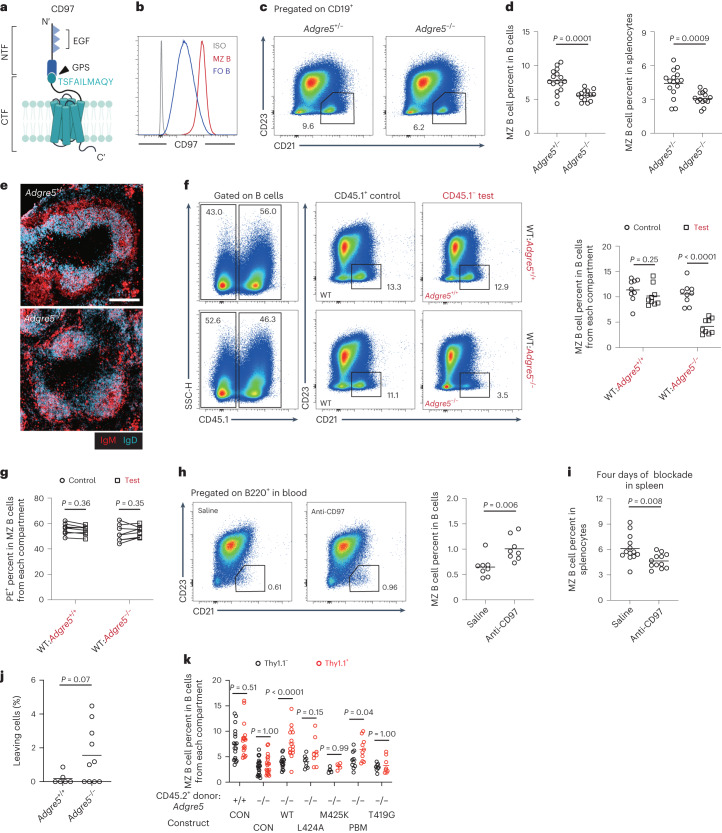
CD97 is required for MZ B cell homeostasis. **a**, Structural components of CD97. Triangles indicate EGF domains. The ten amino acids (419–428) following the GPS are indicated. **b**, Representative histogram plot of CD97 on MZ and follicular (FO) B cells; ISO, isotype control. **c**,**d**, Flow cytometry profiles (**c**) and frequencies of MZ B cells in B cells (**d**; left) and total splenocytes (**d**; right) in *Adgre5*^−/−^ (*n* = 16) and control (*n* = 14) mice. **e**, Distribution of IgM^hi^ MZ (red) and IgD^hi^ follicular (blue) B cells in indicated spleens; scale bar, 200 μm. Sections are representative of multiple cross-sections from at least three mice of each type. **f**, Flow cytometry profiles (left) and frequencies (right) of MZ B cells of the indicated genotypes in WT:*Adgre5*^−/−^ (*n* = 9) and control (*n* = 9) chimeras. **g**, Frequencies of in vivo anti-CD45-phycoerythrin (PE)-labeled MZ B cells of the indicated genotypes in WT:*Adgre5*^−/−^ (*n* = 8) and control (*n* = 8) chimeras. Lines connect data from the same animals. **h**, Flow cytometry profiles (left) and frequencies (right) of MZ B cells in blood in WT mice 3 h after treatment with anti-CD97 (*n* = 8) or saline (*n* = 8). **i**, Frequencies of MZ B cells in the spleen in WT mice after 4 d of treatment with anti-CD97 (*n* = 13) or saline (*n* = 12). **j**, Frequencies of ‘leaving cells’ (GFP^+^ B cells that enter large vessels) in mice reconstituted as in Fig. 1a with *Adgre5*^−/−^ (*n* = 10) and *Adgre5*^+/+^ (*n* = 6) MZ B cells. See corresponding Supplementary Videos 5 and 6. **k**, BM chimeras were reconstituted with 10% non-transduced CD45.1 WT and 90% CD45.2 *Adgre5*^+/+^ or *Adgre5*^−/−^ BM transduced with retroviral constructs encoding *Adgre5* WT (*n* = 15) or its mutants (*n* = 8 in L424A, *n* = 5 in M425K, *n* = 10 in PBM and *n* = 8 in T419G) or empty vector (CON; *n* = 18 in *Adgre5*^+/+^ and *n* = 20 in *Adgre5*^−/−^). The graph shows the frequencies of MZ B cells in Thy1.1^+^ or Thy1.1^−^ B cells. Data are pooled from four (**d** and **i**), two (**f**–**h**), five (**j**) or six (**k**) independent experiments. Each symbol represents one mouse, and lines denote means. Statistical significance was tested by two-tailed *t*-test (**d**, **h**, **i** and **j**) or two-way analysis of variance (ANOVA) followed by a Sidak’s multiple-comparisons test (**f**, **g** and **k**). Source data

**Extended Data Fig. 1:**
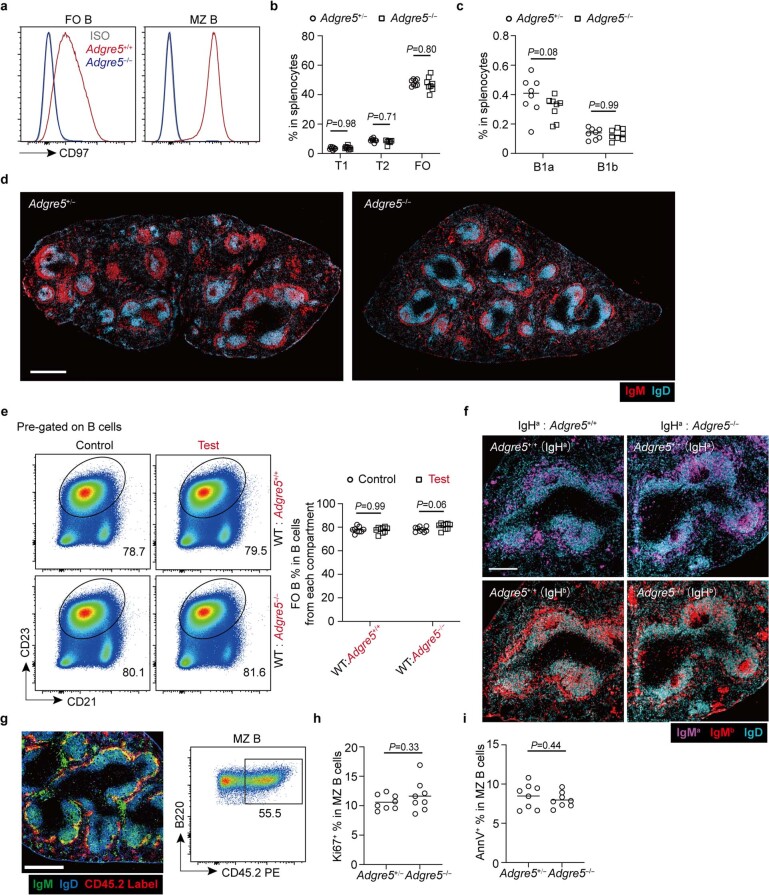
CD97 requirements of splenic MZ B cells. **a**, Representative histogram plot of surface CD97 on FO B (left panel) and MZ B (right panel) cells in *Adgre5*^−/−^ and control mice. **b**, Frequencies of T1, T2 and FO B cells in total splenocytes in *Adgre5*^−/−^ (n = 8) and control (n = 8) mice. **c**, Frequencies of B1a and B1b cells in total splenocytes in *Adgre5*^−/−^ (n = 8) and control (n = 8) mice. **d**, Representative distribution patterns of IgM^hi^ MZ B (red) relative to IgD^hi^ FO B cells (blue) in full spleen cross-sections from mice of the indicated genotypes. Multiple images are stitched to obtain the full spleen view. Scale bar, 500 μm. **e**, Mixed (50:50) bone marrow (BM) chimeras were made with CD45.1 WT (*Adgre5*^+/+^) and CD45.2 *Adgre5*^+/+^ or *Adgre5*^−/−^ BM cells. Representative flow cytometry profiles (left panel) and frequencies (right panel) of FO B cells of indicated genotyped CD19^+^ B cells in WT: *Adgre5*^−/−^ (n = 9) and its control (n = 9) mixed BM chimeras. **f**, Representative distribution patterns of IgM^a^ (magenta) and IgM^b^ MZ B (red) relative to FO B cells (blue) in spleens of 50:50 mixed IgH^a^ WT (*Adgre5*^+/+^) with IgH^b^ *Adgre5*^+/+^ or *Adgre5*^−/−^ chimeras. Scale bar, 200 μm. **g**, Representative distribution patterns (left panel) and frequencies (right panel) of in vivo antibody labeling of MZ B cells (green) relative to FO B cells (blue) in spleens 3 min after i.v. injection of PE-conjugated anti-CD45.2 (red). Blood-exposed MZ B cells (cells that have bound CD45.2-PE) appear yellow. Scale bar, 200 µm. **h**,**i**, Frequencies of Ki-67^+^ (**h**) and Annexin^+^ (**i**) cells in MZ B cells in *Adgre5*^−/−^ (n = 8) and control (n = 8) mice. Data are pooled from two independent experiments (**b**, **c**, **e**, **h**, **i**). Sections are representative of multiple cross-sections from at least three mice (**d**, **f**, **g**). Each symbol represents one mouse and lines denote means. Statistical significance was tested by two-way ANOVA followed by Sidak’s multiple comparisons test (**b**, **c**, **e**) or two-tailed *t*-test (**h**, **i**). Source data

Because frequent interactions were observed between RBCs and MZ B cells, we asked if there were ligand–receptor pairs expressed on the two cell types that might be required for MZ B cell function. Notable among the few surface molecules expressed by RBCs that engage surface receptors is CD55, the decay-accelerating factor of complement that is also a ligand for CD97 (encoded by *Adgre5*)^12,15^. Flow cytometric analysis showed that MZ B cells were marked by high CD97 expression (Fig. 2a,b). We therefore investigated the function of CD97 on MZ B cells. Analysis of CD97-deficient (*Adgre5*^−/−^) mice (Extended Data Fig. 1a) revealed a significant reduction in MZ B cells (Fig. 2c,d). The frequencies of immature (T1 and T2) and mature follicular B cells were unaffected (Extended Data Fig. 1b). The frequency of B1 cells, another type of early-responder B cells^4^, was also unaffected in the spleen (Extended Data Fig. 1c). Immunofluorescence staining of tissue sections for IgM and IgD showed a reduction in the thickness of the IgM^hi^ MZ in mice lacking CD97 (Fig. 2e and Extended Data Fig. 1d). Using mixed bone marrow (BM) chimeras, the CD97 requirement was established to be cell intrinsic (Fig. 2f and Extended Data Fig. 1e). Staining of tissue sections from IgH^a^:IgH^b^ mixed BM chimeras showed a reduction in IgM^hi^ MZ thickness selectively in the CD97-deficient IgM^b^ MZ compartment (Extended Data Fig. 1f). By intravascular antibody labeling of blood-exposed spleen cells, about 55% of MZ B cells in wild-type (WT) mice were labeled; the remaining fraction was within follicles at the time of antibody injection and was protected from labeling^7^ (Extended Data Fig. 1g). CD97 deficiency did not change the fraction of MZ B cells labeled, consistent with the notion that the rapid shuttling of MZ B cells between the MZ and follicle keeps the proportion of cells in each compartment intact even with the overall drop in MZ B cells (Fig. 2g). MZ B cells in littermate-matched control and *Cd97*-KO mice showed the same extent of turnover as determined by staining for the cell cycle antigen Ki-67 (Extended Data Fig. 1h) and the apoptosis marker Annexin V (Extended Data Fig. 1i).

**Extended Data Fig. 2:**
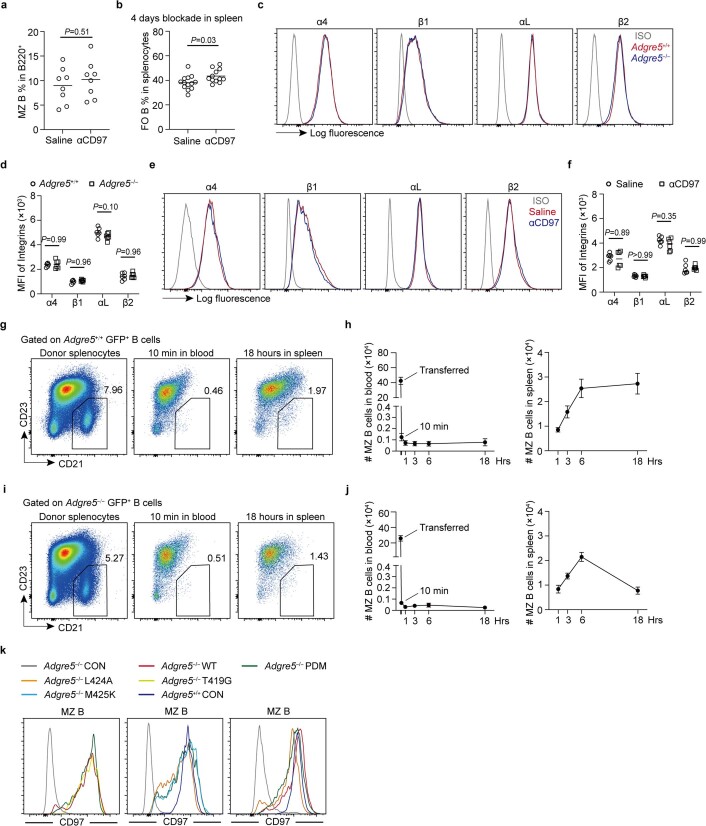
CD97-deficient MZ B cells have disadvantage to re-seed the spleen. **a**, Frequencies of MZ B cells in splenic B220^+^ B cells in WT mice 3 hours treatment with anti-CD97 antibody (n = 8) or saline (n = 8). **b**, Frequencies of FO B cells in spleen of WT mice 4 days after treatment with anti-CD97 antibody (n = 12) or saline (n = 13). **c**,**d**, Representative histogram plots (**c**) and MFI (**d**) of cell surface integrins on MZ B cells in *Adgre5*^−/−^ (n = 6) and control (n = 6) mice. **e**,**f**, Representative histogram plots (**e**) and MFI (**f**) of cell surface integrins in WT mice after 4 days treatment with anti-CD97 antibody (n = 6) or saline (n = 6). **g-j**, *Adgre5*^+/+^ GFP^+^ (**g**, **h**) and *Adgre5*^–/–^ GFP^+^ (**i**, **j**) splenocytes were transferred into WT recipients and the number of donor MZ B cells in blood and spleen determined over time. (**g**,**h**) Representative flow cytometry profiles of MZ B cells among GFP^+^ B cells (**g**) and cell counts (Mean ± SEM) of GFP^+^ MZ B cells staying in blood (**h** left panel) and homing into the spleen (**h** right panel) were analyzed at indicated time points (n = 7 on 0 h, n = 8 on 0.17 h, n = 8 on 1 h, n = 8 on 3 h, n = 8 on 6 h, n = 7 on 18 h). (**i**,**j**) Representative flow cytometry profiles of MZ B cells among GFP^+^ B cells (**i**) and cell counts (Mean ± SEM) of GFP^+^ MZ B cells staying in blood (**j** left panel) and homing into the spleen (**j** right panel) were analyzed at indicated time points (n = 6 on 0 h, n = 8 on 0.17 h, n = 8 on 1 h, n = 8 on 3 h, n = 7 on 6 h, n = 8 on 18 h). The blood volume in all recipients was estimated as 2 ml. **k**, Representative histogram plots of surface CD97 on Thy1.1^+^ CD45.2^+^ MZ B cells in chimeras reconstituted as indicated. See corresponding Fig. 2k. Data are pooled from two (**a**, **d**, **f**, **h**, **j**) or three (**b**) independent experiments. Each symbol represents one mouse and lines denote means. Statistical significance was tested by two-tailed *t*-test (**a**, **b**) or two-way ANOVA followed by Sidak’s multiple comparisons test (**d**, **f**). Source data

We speculated that the reduced frequency of MZ B cells in CD97-deficient mice might be a consequence of loss into blood circulation. However, the frequency of MZ B cells in *Cd97*-KO blood was too low to detect reliably. We considered the possibility that there might be loss of very small numbers of cells at any given moment that could amount to a large loss over periods of days. We therefore asked whether conditional blockade of CD97 ligand binding could lead to a more synchronized release of cells. Indeed, when mice were treated with a CD97-blocking antibody, a small population of MZ B cells could be detected in the blood 3 h later (Fig. 2h). At this time point, the treatment was not sufficient to measurably deplete MZ B cells from the spleen (Extended Data Fig. 2a). However, more prolonged treatment with CD97-blocking antibody led to a reduction in MZ B cells, consistent with gradual loss from the spleen (Fig. 2i). A slight increase in follicular B cell frequency was observed (Extended Data Fig. 2b), likely due to the reduction in MZ B cells.

Intravital two-photon microscopy of the splenic red pulp in mice that harbored GFP^+^ MZ B cells and PKH26^+^ RBCs revealed large numbers of labeled RBCs passing through the red pulp, some at high speed within vessels or sinuses and others more slowly that were likely traveling through the parenchyma (Supplementary Videos 5 and 6). We noted occasional GFP^+^ cells, possibly MZ B cells in the red pulp, in addition to the large GFP^hi^ cells that are likely plasma cells (Supplementary Videos 5 and 6). While imaging spleens of mice that had been reconstituted with either WT or *Cd97*-KO Ub-GFP^+^ B cells, we noted occasional GFP^+^ *Cd97*-KO B cells being released into circulation (Fig. 2j and Supplementary Video 6). Although the trend for release of more *Cd97*-KO cells than WT cells did not reach statistical significance, this observation is consistent with the increased flow cytometric detection of MZ B cells in the blood after CD97 blockade. Integrin expression was intact in CD97-deficient mice and in WT mice after antibody blockade (Extended Data Fig. 2c–f).

To assess the fate of MZ B cells that have been released into blood circulation, we tracked GFP^+^ MZ B cells in the first 18 h after intravenous transfer of splenocytes. Compared to the total number of MZ B cells that were injected, most cells were lost from the blood within 10 min, and only ~0.25% could be recovered 1 h later (Extended Data Fig. 2g,h). Somewhat greater numbers were present in the recipient spleen at this time, and a gradual increase in number occurred, reaching a plateau by 6 h that corresponded to a recovery of about 5% of the transferred MZ B cells. Transferred GFP^+^ CD97-deficient MZ B cells showed a similar loss from circulation and similar initial appearance in the spleen, but these cells were not maintained and had largely decayed by 18 h (Extended Data Fig. 2i,j). Thus, a reduced ability to reseed the spleen may contribute to the overall splenic MZ B cell deficiency in CD97-deficient mice.

### CD97-tethered ligand requirement in MZ B cells

We next characterized what features of CD97 were required for its in vivo function in MZ B cells. CD97 is composed of noncovalently attached NTF and CTF (Fig. 2a). Mutation of T419, the residue immediately following the GPS site, to glycine (T419G) prevents CD97 autoproteolysis but permits normal surface expression^21^. Using a *Cd97*-KO BM retroviral transduction and reconstitution approach, WT CD97 was able to restore MZ B cell accumulation, whereas the non-cleaved T419G mutant did not (Fig. 2k) despite comparable expression (Extended Data Fig. 2k). In these experiments, the transduced CD45.2^+^ cells were detected using a Thy1.1 reporter; untransduced CD45.2^+^ cells in the same BM chimeras were identified as Thy1.1^−^ cells. The first ~10 amino acids following the GPS (Fig. 2a) are thought to function as a tethered ligand in CD97 and many other adhesion GPCRs^12,13^. In a cell line study, mutation of tethered ligand residue L424 to alanine (L424A) or M425 to lysine (M425K) reduced CD97 signaling in vitro^22^. When *Cd97*-KO mice were reconstituted with BM transduced with CD97 L424A or M425K, the MZ B cell compartment was not rescued (Fig. 2k) despite surface expression being comparable to that observed in WT mice (Extended Data Fig. 2k). CD97 has a C-terminal motif that can interact with PDZ domain proteins^21^. CD97 with a mutation in this PDZ-binding motif (PBM) had intact expression (Extended Data Fig. 2k) and was functional in restoring MZ B cells, although perhaps less efficiently than WT CD97 (Fig. 2k). Taken together, these data are consistent with a model where extraction of the CD97 NTF leads to activation of the receptor by a tethered ligand, and this signal promotes MZ B cell retention and homeostasis in the spleen.

### CD97 in MZ B cells signals via Gα_13_ and ARHGEF1

**Fig. 3:**
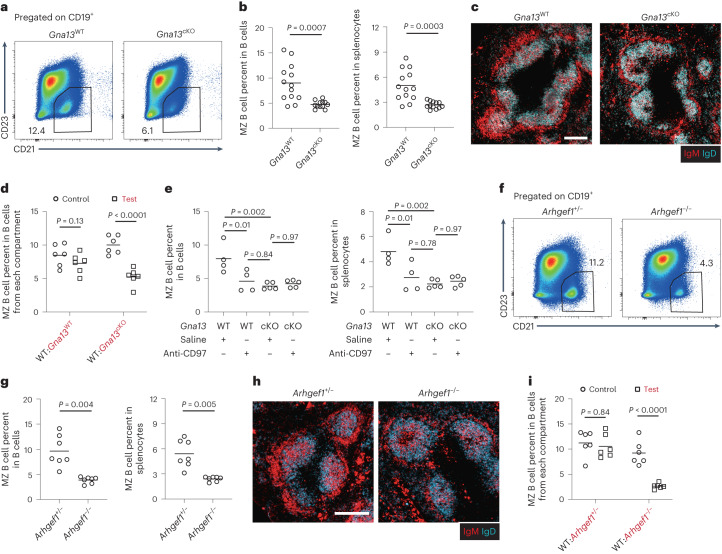
The Gα_13_–ARHGEF1 signaling pathway is required in MZ B cells. **a**,**b**, Representative flow cytometry profiles (**a**) and frequencies of MZ B cells in total CD19^+^ B cells (**b**; left) and in total splenocytes (**b**; right) in *Gna13*^cKO^ (*n* = 12) and control (*n* = 13) mice. **c**, Representative distribution patterns of IgM^hi^ MZ B cells (red) relative to IgD^hi^ follicular B cells (blue) in spleens of mice of the indicated genotypes; scale bar, 200 μm. **d**, Mixed (50:50) BM chimeras were made with CD45.1 WT and CD45.2 *Gna13*^WT^ or *Gna13*^cKO^ BM cells. Frequencies of MZ B cells among the indicated genotype CD19^+^ B cells in WT:*Gna13*^cKO^ (*n* = 6) and control (*n* = 6) mixed BM chimeras. **e**, Frequencies of MZ B cells in total CD19^+^ B cells (left) and in total splenocytes (right) in *Gna13*^cKO^ (*n* = 5) and control (*n* = 4) mice 4 d after treatment with anti-CD97 or saline. **f**,**g**, Representative flow cytometry profiles (**f**) and frequencies of MZ B cells in total CD19^+^ B cells (**g**; left) and in total splenocytes (**g**; right) in *Arhgef1*^−/−^ (*n* = 7) and control (*n* = 7) mice. **h**, Representative distribution patterns of IgM^hi^ MZ B cells (red) relative to IgD^hi^ follicular B cells (blue) in spleens of mice of the indicated genotypes; scale bar, 200 μm. **i**, Mixed (50:50) BM chimeras were made with CD45.1 WT and CD45.2 *Arhgef1*^+/−^ or *Arhgef1*^−/−^ BM cells. Frequencies of MZ B cells among the indicated genotype CD19^+^ B cells in WT:*Arhgef1*^−/−^ (*n* = 6) and control (*n* = 6) mixed BM chimeras. Data are pooled from four (**b**) or two (**d**, **e**, **g** and **i**) independent experiments. Sections are representative of multiple cross-sections from at least three mice of each type (**c** and **h**). Each symbol represents one mouse, and lines denote means. Statistical significance was tested by two-tailed *t*-test (**b** and **g**) or two-way ANOVA followed by a Sidak’s multiple-comparisons test (**d** and **i**) or one-way ANOVA followed by a Tukey’s multiple-comparisons test (**e**). Source data

**Extended Data Fig. 3:**
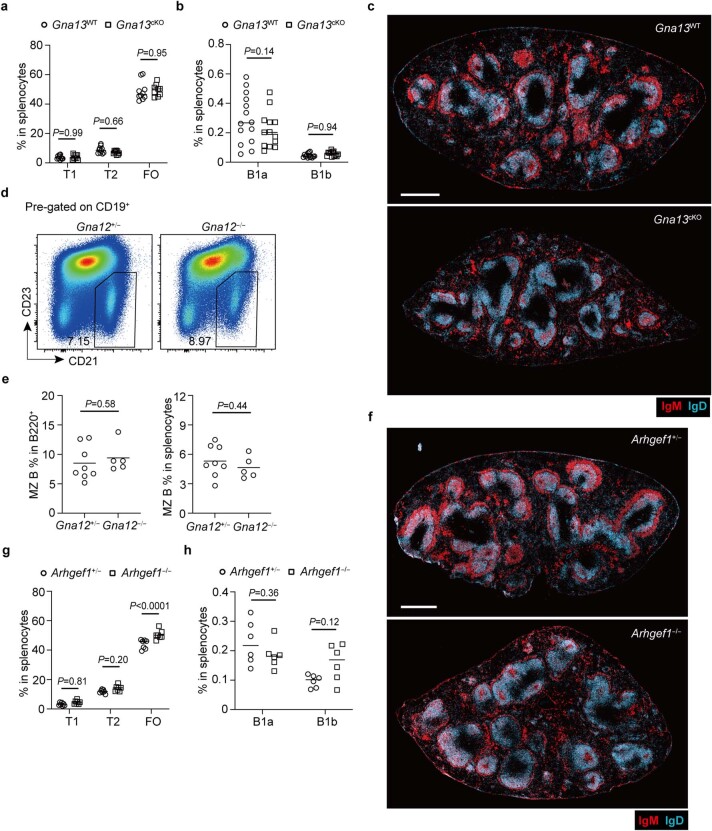
Selective Gα13-ArhGEF1 requirements of splenic B cells. **a**, Frequencies of T1, T2 and FO B cells in total splenocytes in *Gna13*^cKO^ (n = 9) and control (n = 10) mice. **b**, Frequencies of B1a and B1b cells in total splenocytes in *Gna13*^cKO^ (n = 13) and control (n = 14) mice. **c**, Representative distribution patterns of IgM^hi^ MZ B (red) relative to IgD^hi^ FO B cells (blue) in full spleen cross-sections from mice of the indicated genotypes. Scale bar, 500 μm. **d**,**e**, Representative flow cytometry profiles (**d**) and frequencies of MZ B in total CD19^+^ B cells (**e** left panel), and in total splenocytes (**e** right panel) in *Gna12*^−/−^ (n = 8) and control (n = 5) chimeras. **f**, Representative distribution patterns of IgM^hi^ MZ B (red) relative to IgD^hi^ FO B cells (blue) in full spleen cross-sections from mice of the indicated genotypes. Scale bar, 500 μm. **g**, Frequencies of T1, T2 and FO B cells in total splenocytes in *Arhgef1*^−/−^ (n = 7) and control (n = 7) mice. **h**, Frequencies of B1a and B1b cells in total splenocytes in *Arhgef1*^−/−^ (n = 6) and control (n = 6) mice. Data are pooled from two (**a**, **e**, **g**, **h**) or three (**b**) independent experiments. Sections are representative of multiple cross-sections from at least three mice of each type (**c**, **f**). Each symbol represents one mouse and lines denote means. Statistical significance was tested by two-way ANOVA followed by Sidak’s multiple comparisons test (**a**, **b**, **g**, **h**) or two-tailed *t*-test (**e**). Source data

Because prior studies in cell lines and type 2 conventional dendritic cells indicated that CD97 can signal via Gα_13_-containing heterotrimeric G proteins^17,21,23,24^, and other work showed a role for Gα_12_/Gα_13_ in MZ B cells^25^, we asked if Gα_13_ may be required for CD97 function in MZ B cells. Like CD97-deficient mice, animals lacking Gα_13_ selectively in B cells (*Gna13*^fl/fl^ Mb1-Cre^+^ conditional knockout (cKO), labeled as *Gna13*^cKO^) showed a twofold reduction in MZ B cell frequency (Fig. 3a,b). There was no effect of Gα_13_ deficiency on immature or mature B cell frequencies in the spleen (Extended Data Fig. 3a). Splenic B1 cells were also unaffected (Extended Data Fig. 3b). Microscopy showed that *Gna13*^cKO^ mice had reduced MZ thickness (Fig. 3c and Extended Data Fig. 3c). Using mixed BM chimeras, the Gα_13_ requirement for MZ B cell homeostasis was confirmed to be cell intrinsic (Fig. 3d). Importantly, chronic anti-CD97 treatment of Gα_13_-deficient mice did not cause any further reduction in MZ B cell frequencies, whereas it did reduce the MZ B cell compartment in WT mice, as expected (Fig. 3e). Gα_12_ deficiency did not affect MZ B cell frequencies (Extended Data Fig. 3d,e). These data confirm that CD97 and Gα_13_ function in the same pathway. ARHGEF1 (also known as p115RhoGEF or Lsc) is a Rho-activating guanidine nucleotide exchange factor and the best-defined effector of Gα_13_ (ref. ^26^). A similar series of experiments performed with ARHGEF1-deficient mice showed that this Gα_13_ effector is needed for MZ B cell homeostasis (Fig. 3f–h and Extended Data Fig. 3f), in agreement with a prior study^27^. ARHGEF1 deficiency did not affect immature B cell or B1 cell frequencies in the spleen, and it led to a slight increase in follicular B cell frequencies (Extended Data Fig. 3g,h). Mixed BM chimeras established that ARHGEF1 acts in a cell-intrinsic manner in MZ B cells (Fig. 3i). These findings are in agreement with CD97 signaling via Gα_13_ and ARHGEF1 in MZ B cells.

### CD55 on RBCs acts as the CD97 ligand

**Fig. 4:**
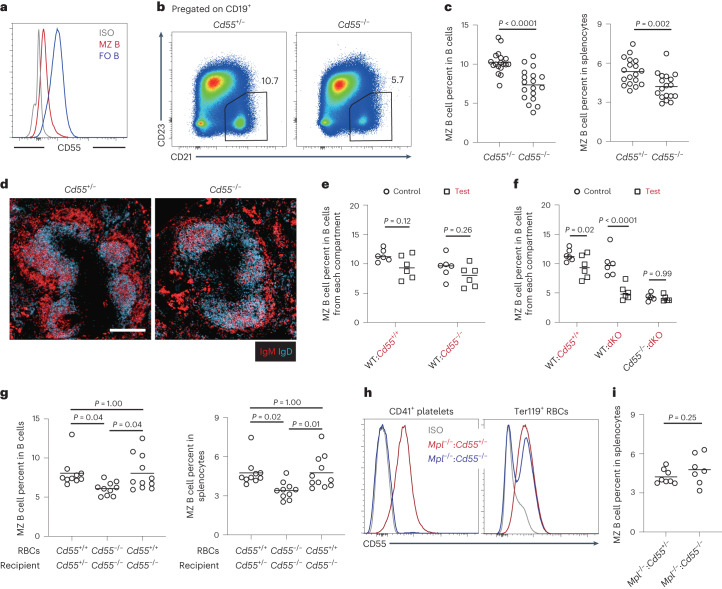
CD55 on RBCs is required for MZ B cell maintenance. **a**, Representative histogram plot of surface CD55 on MZ B cells and follicular B cells. **b**,**c**, Representative flow cytometry profiles (**b**) and frequencies of MZ B cells in total CD19^+^ B cells (**c**; left) and in total splenocytes (**c**; right) in *Cd55*^−/−^ (*n* = 18) and control (*n* = 18) mice. **d**, Representative distribution patterns of IgM^hi^ MZ B cells (red) relative to IgD^hi^ follicular B cells (blue) in spleens of mice of the indicated genotypes; scale bar, 200 μm. Sections are representative of multiple cross-sections from at least three mice of each type. **e**, Mixed (50:50) BM chimeras were made with CD45.1 WT and CD45.2 *Cd55*^+/+^ or *Cd55*^−/−^ BM cells. Frequencies of MZ B cells of the indicated genotype CD19^+^ B cells in WT:*Cd55*^−/−^ (*n* = 6) and control (*n* = 6) mixed BM chimeras. **f**, Mixed (50:50) BM chimeras were made with CD45.1 WT and CD45.2 WT (*n* = 6), *Adgre5*^−/−^*Cd55*^−/−^ (dKO; *n* = 6) or *Cd55*^−/−^ and dKO (*n* = 5) BM cells. Frequencies of MZ B cells among the indicated genotype CD19^+^ B cells in mixed BM chimeras. **g**, RBC transfusions were performed from *Cd55*^−/−^ or *Cd55*^+/+^ mice to the indicated recipient mice once per week, and analysis was performed after 4 weeks. Frequencies of MZ B cells in total B cells (left) and in total splenocytes (right) in mice with purified RBCs transfused as indicated (*n* = 10 or 11 recipients in each group) are shown. **h**,**i**, Mixed chimeras were made with 85% *Mpl*^−/−^ and 15% *Cd55*^+/+^ or *Cd55*^−/−^ BM cells. A representative histogram shows surface CD55 on platelets (left) and RBCs (right) in chimeras (**h**). Frequencies of MZ B cells in total splenocytes in *Mpl*^−/−^:*Cd55*^−/−^ (*n* = 7) and control (*n* = 8) chimeras are shown (**i**). Data are pooled from five (**c**), two (**e**, **f** and **i**) or three (**g**) independent experiments. Each symbol represents one mouse, and lines denote means. Statistical significance was tested by two-tailed *t*-test (**c** and **i**) or two-way ANOVA followed by Sidak’s multiple-comparisons test (**e** and **f**) or one-way ANOVA followed by Tukey’s multiple-comparisons test (**g**). Source data

**Extended Data Fig. 4:**
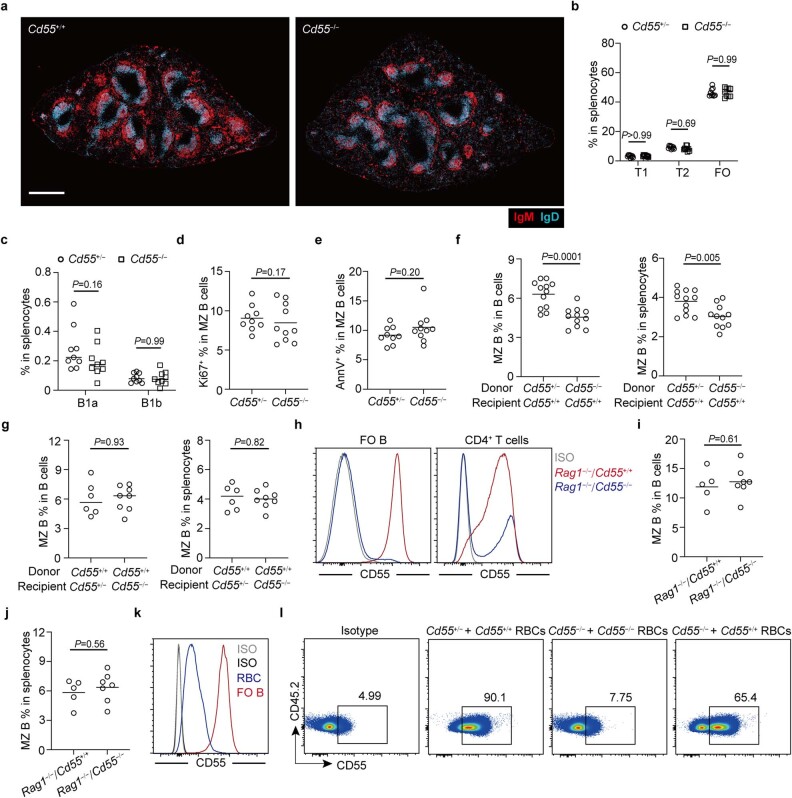
CD55 requirements of splenic MZ B cells. **a**, Representative distribution patterns of IgM^hi^ MZ B (red) relative to IgD^hi^ FO B cells (blue) in full spleen cross-sections from mice of the indicated genotypes. Scale bar, 500 μm. Sections are representative of multiple cross-sections from at least three mice of each type. **b**, Frequencies of T1, T2 and FO B cells in total splenocytes in *Cd55*^−/−^ (n = 9) and control (n = 9) mice. **c**, Frequencies of B1a and B1b cells in total splenocytes in *Cd55*^−/−^ (n = 9) and control (n = 9) mice. **d**,**e**, Frequencies of Ki-67^+^ (**d**) and Annexin^+^ (**e**) cells in MZ B cells in *Cd55*^−/−^ (n = 10) and control (n = 9) mice. **f**, Frequencies of MZ B cells in total CD19^+^ B cells (left panel), and in total splenocytes (right panel) in *Cd55*^−/−^ (n = 11) and control (n = 12) chimeras as indicated. **g**, Frequencies of MZ B cells in total CD19^+^ B cells (left panel), and in total splenocytes (right panel) in *Cd55*^−/−^ (n = 8) and control (n = 6) reverse chimeras as indicated. **h**-**j**, Chimeras reconstituted with 85% *Rag1*^−/−^ and 15% *Cd55*^+/+^ or *Cd55*^−/−^ BM cells. (**h**) Representative histogram plot of surface CD55 on FO B cells (left panel) and CD4 T cells (right panel) in chimeras as indicated. (**i**,**j**) Frequencies of MZ B cells in total CD19^+^ B cells (**i**), and in total splenocytes (**j**) in *Rag1*^−/−^: *Cd55*^−/−^ (n = 7) and its control (n = 5) chimeras. **k**, Representative histogram plot of surface CD55 on FO B cells and RBCs. **l**, Representative profile of purified *Cd55*^+/+^ RBCs or *Cd55*^−/−^ RBCs transfused into the indicated recipient mice once per week for 4 weeks. Data are pooled from two (**b**-**e**, **g**, **i**, **j**) or three (**f**) independent experiments. Each symbol represents one mouse and lines denote means. Statistical significance was tested by two-way ANOVA followed by Sidak’s multiple comparisons test (**b**, **c**) or two-tailed *t*-test (**d**-**g**, **i**, **j**). Source data

CD55 is well expressed on RBCs and is also present on various other hematopoietic cells^15,17^. Interestingly, MZ B cells expressed low amounts of CD55 compared to follicular B cells (Fig. 4a), perhaps ensuring limited *cis* interaction between CD55 and CD97 and maximal availability to interact with CD55 on other cells. To test the importance of CD55 for CD97-dependent functions in MZ B cells, we analyzed *Cd55*^−/−^ mice. MZ B cell frequencies were reduced in *Cd55*^−/−^ mice to a similar extent as in *Cd97*-KO mice, and the MZ showed a similar decrease in thickness in imaging (Fig. 4b–d and Extended Data Fig. 4a). Immature, follicular and B1 cell frequencies in the spleen were unaltered by CD55 deficiency (Extended Data Fig. 4b,c). The fractions of MZ B cells in the cell cycle based on Ki-67 staining (Extended Data Fig. 4d) or that were undergoing cell death based on Annexin V staining (Extended Data Fig. 4e) were not changed by CD55 deficiency. Mixed BM chimeras showed that CD55 was not required intrinsically by MZ B cells (Fig. 4e). Importantly, when cells lacked both CD55 and CD97 (double KO (dKO)), the deficiency in MZ B cells was of the same magnitude as for single-KO cells (Fig. 4f), consistent with these genes acting in the same pathway and with CD55 serving as the only CD97 ligand involved in MZ B cell maintenance.

In addition to being expressed by RBCs and some other hematopoietic cells, CD55 is expressed by various non-hematopoietic cells^17,28^. As a broad approach to determine the necessary CD55-expressing cell types for MZ B cell homeostasis, we generated reciprocal BM chimeras. In mice lacking CD55 in all radiation-sensitive BM-derived cells, there was a deficiency in MZ B cells (Extended Data Fig. 4f), whereas in mice lacking CD55 in radiation-resistant cells, including all stromal cells, the MZ B cell compartment remained intact (Extended Data Fig. 4g). Although follicular B cells abundantly express CD55, analysis of 85:15 *Rag1*^−/−^:*Cd55*^−/−^ BM chimeras that lack CD55 on all B cells and most T cells revealed an intact MZ B cell compartment (Extended Data Fig. 4h–j). RBCs make up 99.9% of the cells in blood^29^. RBCs also express CD55, although at a lower surface level than B cells (Extended Data Fig. 4k). To test whether RBCs were the relevant source of CD55, WT or *Cd55*-KO RBCs were transferred into *Cd55*-KO mice. Because the MZ B cell compartment is slow to turn over^4^, the transfusion was performed weekly for 4 weeks. At the end of the transfusion, approximately 65% of RBCs were of donor origin (Extended Data Fig. 4l). Reconstitution of *Cd55*^−/−^ mice with WT, but not *Cd55*-KO, RBCs rescued the size of the MZ B cell compartment (Fig. 4g). Platelets also express CD55 (ref. ^30^). To test for a possible contribution of platelet CD55 to MZ B cell homeostasis, 85:15 mixed BM chimeras between *Mpl*^−/−^ BM (unable to generate platelets) and *Cd55*^−/−^ BM were made such that all the platelets in these mice were CD55 deficient (Fig. 4h). CD55 deficiency on platelets did not lead to a reduction in the MZ B cell compartment (Fig. 4i). Thus, RBCs are the key CD55^+^ cell type needed for MZ B cell homeostasis.

### CD55-dependent extraction of the CD97 NTF under shear stress

**Fig. 5:**
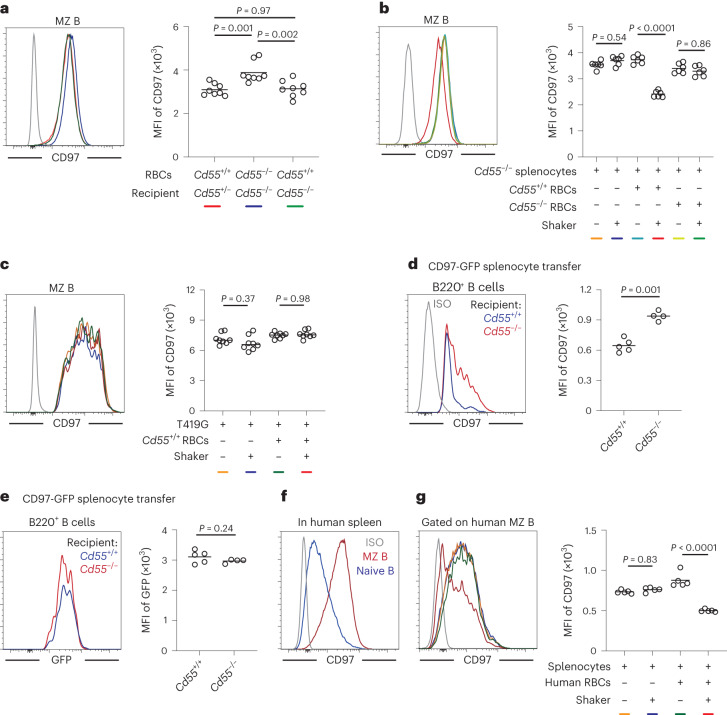
CD55-mediated CD97 NTF extraction is dependent on shear stress and is conserved in human MZ B cells. **a**, Representative histogram (left) and geometric mean fluorescence intensity (MFI; right) of surface CD97 expression on MZ B cells in mice with blood transfusion as indicated (*n* = 8 recipients in each group). Line colors in the histogram are for mice of the types shown by color code beneath the graph. **b**,**c**, Splenocytes from *Cd55*^−/−^ mice (**b**; *n* = 6 in each group) or CD97 T419G-expressing BM chimeras (**c**; *n* = 8 in each group) were cocultured for 45 min with *Cd55*^−/−^ or *Cd55*^+/+^ RBCs on a shaker or not. Representative histograms (left) and MFIs (right) of surface CD97 expression on MZ B cells are shown. Line colors in the histograms are for conditions of the types shown by color codes beneath the graphs. Each symbol represents one incubation, and each donor contributes two incubations. **d**,**e**, MFI of surface CD97 expression (**d**) and GFP (**e**) on transferred MZ B cells from CD97–GFP fusion protein-expressing BM chimeras in blood collected from *Cd55*^−/−^ (*n* = 4) or *Cd55*^+/+^ (*n* = 5) recipients after a 30-min transfer as indicated. Each symbol represents one mouse. **f**, Representative histogram plot of surface CD97 on naive B cells and MZ B cells from the human spleen. **g**, Representative histogram (left) and MFI (right) of surface CD97 expression on human MZ B cells after incubation for 45 min with human RBCs on a shaker or not (*n* = 5 in each group). Each symbol represents one incubation, and line colors in the histogram are for conditions of the types shown by color code beneath the graph. Lines in graph denote means. Data are pooled from two independent experiments (**a**–**e**) or are representative of two independent experiments (**g**). Statistical significance was tested by one-way ANOVA followed by Tukey’s multiple-comparisons test (**a**–**c** and **g**) or two-tailed *t*-test (**d** and **e**). Source data

**Extended Data Fig. 5:**
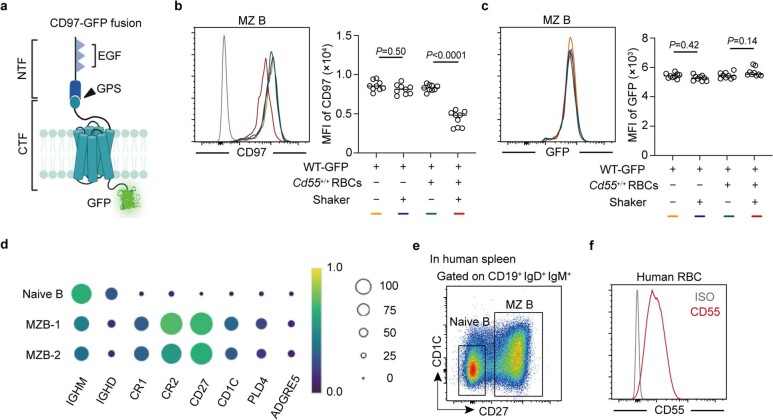
CD55-mediated extraction of CD97 NTF under shear stress is conserved on human MZ B cells. **a**, Structural components of the CD97-GFP fusion protein. NTF, N-terminal fragment; CTF, C-terminal fragment; GPS, GPCR proteolysis site; triangles indicate individual epidermal growth factor (EGF) domains; GFP, Green fluorescent protein. **b**,**c**, Splenocytes from CD97-GFP fusion protein expressing BM chimeras were cocultured for 45 minutes at 24 °C with *Cd55*^+/+^ RBCs, on a shaker or not. Representative histogram (left panel) and MFI (right panel) of surface CD97 expression (**b**) and GFP (**c**) on MZ B cells (n = 9 in each group). Data are pooled from two independent experiments. Each symbol represents one incubation and each donor contributes three incubations. Lines denote means, and line colors in histograms are for conditions of the types shown by color codes beneath the graphs. Statistical significance was tested by one-way ANOVA followed by Tukey’s multiple comparisons test. **d**, Dot plot illustrating marker gene expression for human specific B cell subtypes. Data are from GSE193869 (ref. ^33^). **e**, Representative flow cytometry profiles displaying gates of human naïve and MZ B cells. **f**, Representative histogram plot of surface CD55 on human peripheral RBCs. Source data

Flow cytometric analysis of MZ B cells for CD97 showed that surface levels were elevated in mice lacking CD55 (Fig. 5a) and returned to normal in mice receiving transfers of RBCs from WT donor mice (Fig. 5a). Under in vitro conditions, MZ B cell CD97 surface abundance was reduced after 45 min of co-incubation with WT RBCs in a shaker at 1,000 r.p.m., which generates a shear stress of approximately 14 dyne cm^−2^ (ref. ^31^; Fig. 5b). MZ B cells incubated with RBCs without shaking or incubated with *Cd55*^−/−^ RBCs with shaking did not show any reduction in CD97 surface abundance (Fig. 5b). When MZ B cells were taken from CD97-deficient mice that had been reconstituted with BM transduced with a construct encoding the non-cleavable T419G form of CD97, incubation with RBCs with shaking did not lead to any change in CD97 surface abundance, consistent with the reduced expression being due to extraction of the NTF (Fig. 5c). To confirm that exposure to CD55^+^ RBCs under shear stress was causing NTF extraction and not CD97 degradation, we used MZ B cells from chimeras that had been reconstituted with a CD97–GFP fusion protein (Extended Data Fig. 5a). Exposure of MZ B cells expressing this construct to *Cd55*^+/+^ RBCs under shear stress conditions led to reduced CD97 surface staining but had no effect on GFP intensity (Extended Data Fig. 5b,c), confirming that the reduced staining was due to extraction of the NTF. Finally, we transferred splenic B cells from chimeras expressing the CD97–GFP fusion protein into WT or *Cd55*-KO recipients and analyzed the transferred cells in recipient blood 30 min later. Due to the rarity of transferred MZ B cells in the recipient blood, we instead tracked changes in CD97 and GFP abundance in transferred follicular B cells. Compared to B cells in *Cd55*-KO recipients, B cells in the blood of WT recipients had low CD97 surface staining, but GFP intensity was unaffected (Fig. 5d,e). Taken together, these data provide evidence that CD97 on MZ B cells undergoes NTF extraction following encounter with CD55^+^ RBCs under shear stress conditions.

Human splenic MZ B cells are CD27^hi^, and they have variable levels of CD1c^32,33^. Analysis of published single-cell RNA-sequencing data of human spleen B cells showed higher *ADGRE5* mRNA expression in the two MZ B cell clusters, although expression of transcripts did not appear abundant compared to that for CD27 or CR2 (Extended Data Fig. 5d)^33^. However, fluorescence-activated cell sorting analysis established that most MZ B cells express higher amounts of CD97 protein than follicular B cells (Fig. 5f and Extended Data Fig. 5e). Human RBCs were positive for CD55 expression (Extended Data Fig. 5f), as expected^30^. When RBC-depleted human splenocytes were incubated in the presence of human RBCs under shear stress, there was a marked reduction in CD97 surface staining compared to splenocytes incubated without RBCs or without shear stress (Fig. 5g). These data suggest that human MZ B cells experience CD97 NTF extraction during interactions with RBCs under shear stress conditions.

### CD97 promotes cell membrane retraction

**Fig. 6:**
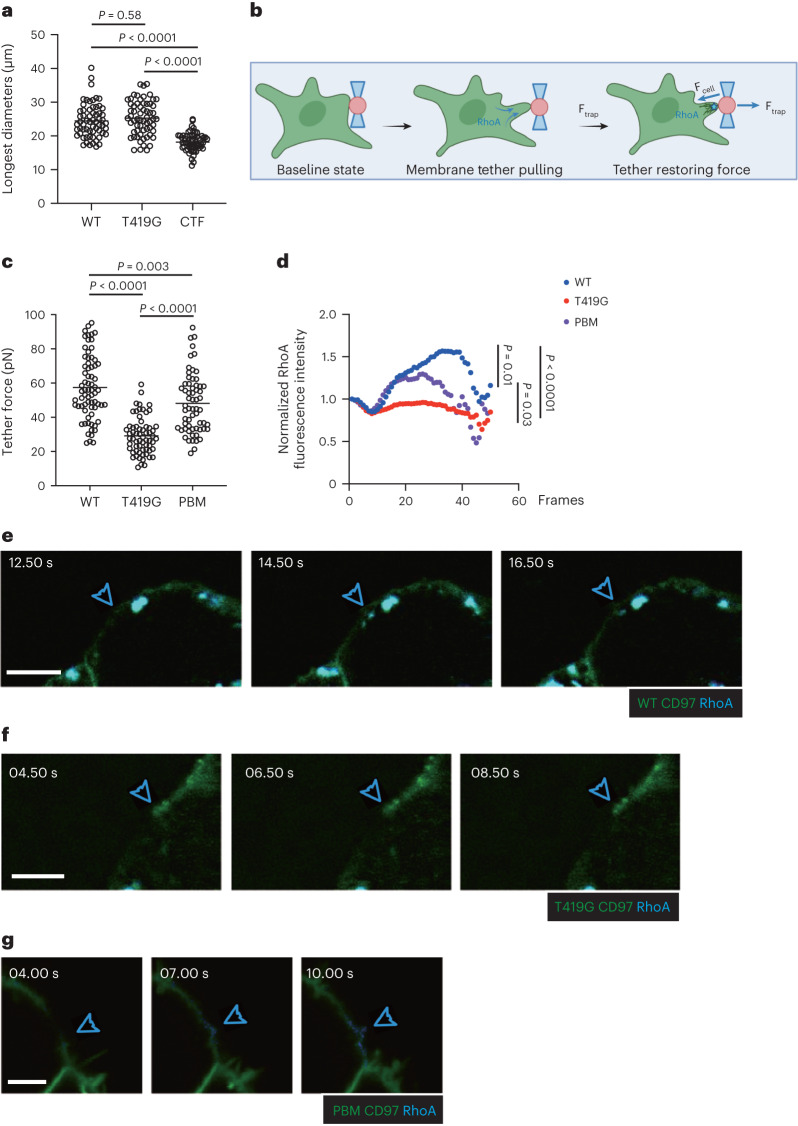
CD97 promotes cell membrane retraction through activation of RhoA. **a**, HEK293T cells were transfected with CD97 (WT)–GFP, CD97 (T419G)–GFP or CTF–GFP fusion constructs. Longest diameters of individual WT- (*n* = 62), T419G- (*n* = 62) and CTF-transfected (*n* = 63) cells are shown. Each symbol represents one cell. One of three independent experiments with similar results is shown. **b**, Schematic diagram of an optically trapped ligand-coated bead applying pulling force while in contact with the cell membrane. The cell is also indicated to contain an anillin–GFP RhoA biosensor. **c**,**d**, HEK293T cells were cotransduced with anillin–GFP RhoA biosensor and CD97 (WT)–Scarlet, CD97 (T419G)–Scarlet or CD97 (PBM)–Scarlet fusion lentivirus and sorted. **c**, Cell trap forces of individual WT- (*n* = 70), T419G- (*n* = 64) and PBM-transduced (*n* = 62) cells. **d**, Normalized active RhoA biosensor fluorescence intensity of WT- (*n* = 27), T419G- (*n* = 20) and PBM-transduced (*n* = 32) cells as indicated. **e**–**g**, Examples of anillin–GFP active RhoA biosensor signals at the site of bead contact and membrane tether (blue arrowhead) of one HEK293T cell expressing CD97 (WT)–GFP fusion (**e**), CD97 (T419G)–GFP fusion (**f**) or CD97 (PBM)–GFP fusion (**g**) protein. The blue arrowhead indicates the site of bead pulling. The time series begins at the start time for pulling, which was about 30 s after the bead contacted the cell. See corresponding Supplementary Videos 7–12; scale bar, 5 μm (**e** and **f**); scale bar, 3 μm (**g**). Data are representative of three independent experiments (**a**) or a pool from two (**c**–**g**) independent experiments. Statistical significance was tested by one-way ANOVA followed by a Tukey’s multiple-comparisons test (**a** and **c**) or mixed-effects analysis followed by a Geisser–Greenhouse correction (**d**). Source data

**Extended Data Fig. 6:**
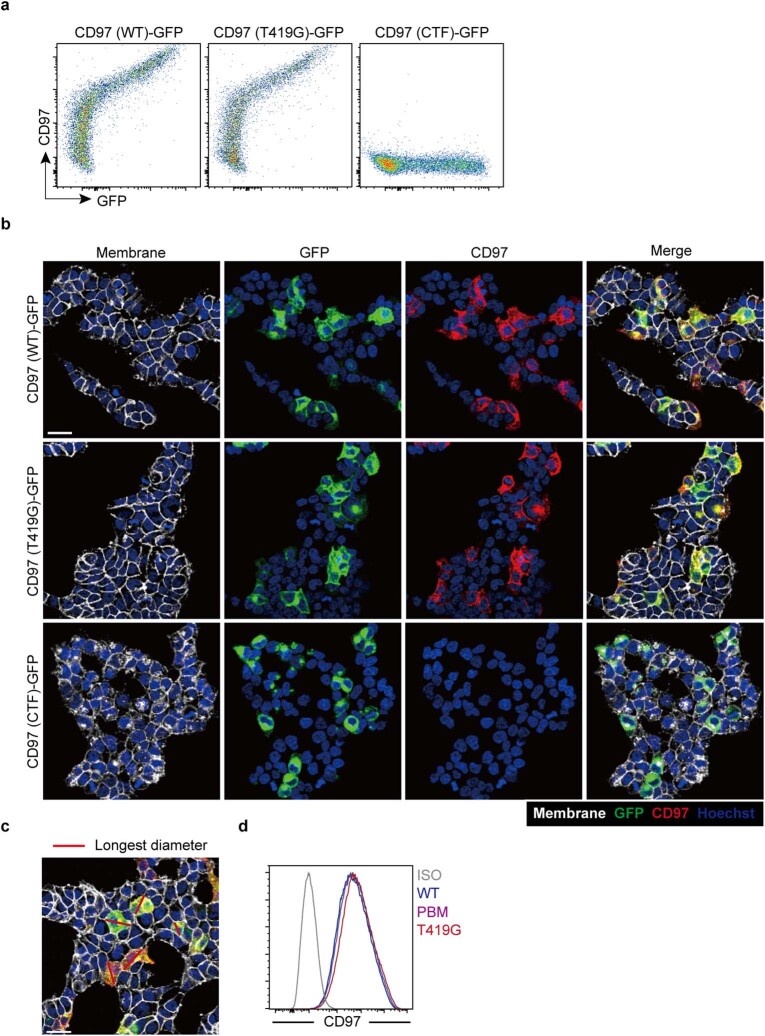
CD97 promotes cell membrane retraction through activation of RhoA. **a**-**c**, HEK293T cells were transfected with CD97 (WT)-GFP, CD97 (T419G)-GFP, or CTF-GFP fusion constructs. (**a**) Representative flow cytometry profiles of CD97 and GFP in transfected HEK293T cells 40 hours post transfection. (**b**) Cell membranes, nucleus, GFP and surface CD97 were stained and imaged. Scale bar, 30 μm. Data are representative of three independent experiments. (**c**) Longest diameters of transfected individual cells were measured with ImageJ. **d**, HEK293T cells were co-transduced with Anillin-GFP RhoA Biosensor and CD97 (WT)-Scarlet, CD97 (T419G)-Scarlet, or CD97 (PBM)-Scarlet fusion lentivirus and sorted. Representative histogram plot of surface CD97 on sorted HEK293T cells.

Activation of Rho-based signaling is established to promote retraction of cell membrane processes, and there is in vitro evidence of CD97 activation of Rho^21,23,34^. However, it remains unclear whether shear stress-mediated activation of CD97 is sufficient to engage Rho activity and membrane retraction. We examined the impact of CD97 expression (Extended Data Fig. 6a) on the morphology of adherent HEK293T cells. Cells expressing WT CD97 or the cleavage-resistant T419G CD97 variant showed a similar irregular morphology (Extended Data Fig. 6b). By contrast, cells expressing the CD97 CTF were more rounded (Extended Data Fig. 6b). The differences in shape were confirmed by comparing the longest dimension of the cells under each condition (Fig. 6a and Extended Data Fig. 6c).

To directly test whether the CD55–CD97 interaction leads to membrane retraction, we turned to optical tweezers^35^. Beads coated with recombinant mouse CD55 were brought into contact with HEK293T cells expressing mouse CD97–Scarlet for 30 s to allow a membrane tether attachment. Subsequently, the bead was pulled away using the optical trap^35^. The displaced bead remains attached to the cell via a membrane tether, which exerts a restoring force that is proportional to the square of membrane tension^35,36^ (Fig. 6b). These measurements revealed that cells expressing WT CD97 exerted an almost twofold greater tether force than cells expressing the signaling defective T419G CD97 variant (Fig. 6c and Extended Data Fig. 6d). Similar analysis of the CD97 PBM variant mutated in the C-terminal PBM showed a partial reduction in the retraction response (Fig. 6c and Extended Data Fig. 6d). Using cells that also expressed the active RhoA biosensor anillin^37^ and WT CD97, induction of RhoA activation at the bead contact site was observed within a few seconds after applying a pulling force on the bead (Fig. 6d,e and Supplementary Videos 7 and 8). RhoA activation was not observed at the bead contact site in equivalently treated cells that expressed the T419G CD97 mutant (Fig. 6d,f and Supplementary Videos 9 and 10). RhoA activation was induced by the CD97 PBM mutant, although the activity was less sustained than for WT CD97, suggesting that there is a difference in the kinetics of recruitment and activation of RhoA (Fig. 6d,g and Supplementary Videos 11 and 12). These data are consistent with CD97 becoming activated to signal via RhoA and cause membrane retraction after binding CD55^+^ particles and exposure to pulling forces.

### CD97 deficiency leads to reduced T cell-independent responses

**Fig. 7:**
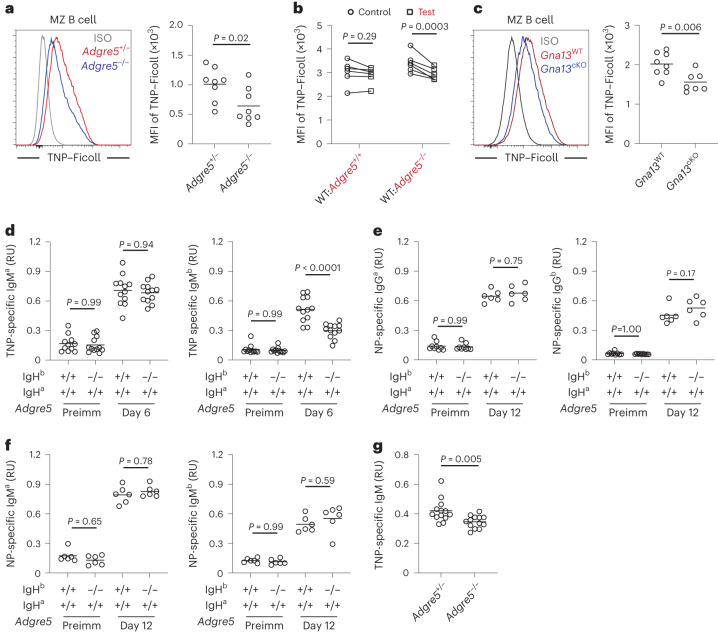
CD97 deficiency leads to defective antibody responses to T cell-independent antigen. **a**–**c**, Mice were analyzed 40 min after intravenous TNP–Ficoll injection. **a**, Representative histogram (left) and MFI (right) of TNP–Ficoll levels on MZ B cells in *Adgre5*^−/−^ (*n* = 8) and control (*n* = 8) mice. **b**, MFI of TNP–Ficoll levels on MZ B cells in WT:*Adgre5*^−/−^ (*n* = 6) and control (*n* = 6) mixed BM chimeras. Lines connect data from the same animals. **c**, Representative histogram (left) and MFI (right) of TNP–Ficoll levels on MZ B cells in *Gna13*^cKO^ (*n* = 8) and control (*n* = 7) mice. **d**–**f**, Enzyme-linked immunosorbent assay (ELISA) analysis of serum from 50:50 mixed IgH^a^ WT (*Adgre5*^+/+^) with IgH^b^ *Adgre5*^+/+^ or *Adgre5*^−/−^ chimeras. **d**, Mixed chimeras were immunized intravenously with TNP–Ficoll. TNP-specific IgM^a^ (left) and IgM^b^ (right) in serum from the indicated time points (*n* = 11 or 12 chimeras for each condition). **e**,**f**, Mixed chimeras were immunized intraperitoneally with NP-CGG with Alum. NP-specific IgG1^a^ (**e**; left), IgG1^b^ (**e**; right; *n* = 9 for preimmunized (preimm) and *n* = 6 for postimmunized groups), IgM^a^ (**f**; left) and IgM^b^ (**f**; right; *n* = 6 for each group) in serum from the indicated time points. **g**, TNP-specific IgM in serum from *Adgre5*^+/−^ (*n* = 13) or *Adgre5*^−/−^ (*n* = 13) mice day 5 after TNP–Ficoll immunization. In **d**–**g**, RU indicates relative units. Data are pooled from two (**a**–**c**, **e** and **f**) or three (**d** and **g**) independent experiments. Each symbol represents one mouse, and lines denote means. Statistical significance was tested by two-tailed *t*-test (**a**, **c** and **g**) or two-way ANOVA followed by a Sidak’s multiple-comparisons test (**b**) or one-way ANOVA followed by a Tukey’s multiple-comparisons test (**d**–**f**). Source data

**Extended Data Fig. 7:**
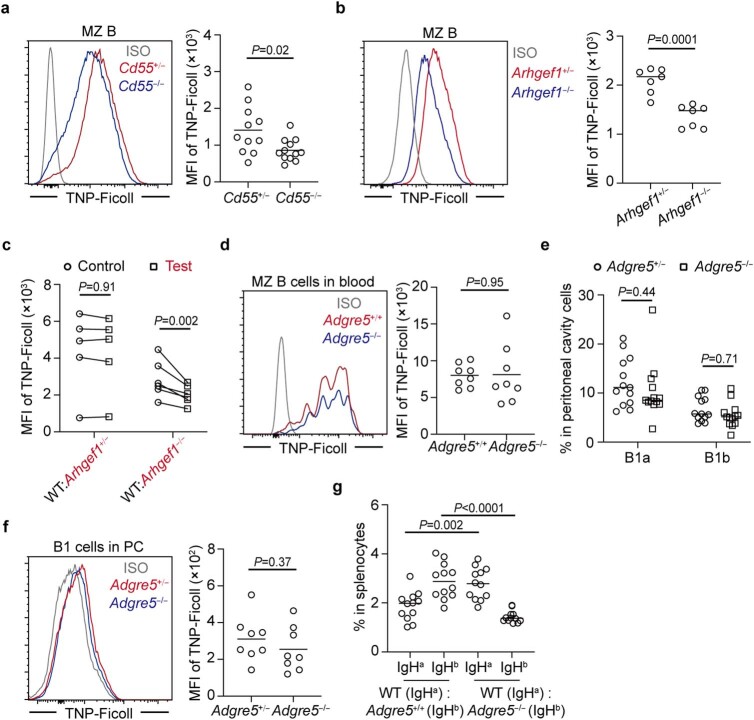
CD97-pathway deficiency leads to defective early antibody responses to T-independent antigen. **a**-**c**, Mice were analyzed 40 minutes after intravenous TNP-Ficoll injection. (**a**) Representative histogram (left panel) and MFI (right panel) of TNP-Ficoll level on MZ B cells in *Cd55*^−/−^ (n = 12) and control (n = 11) mice. (**b**) Representative histogram (left panel) and MFI (right panel) of TNP-Ficoll level on MZ B cells in *Arhgef1*^−/−^ (n = 7) and control (n = 7) mice. (**c**) MFI of TNP-Ficoll level on MZ B cells in WT:*Arhgef1*^−/−^ (n = 6) and its control (n = 5) mixed BM chimeras. Lines connect data from same animals. **d**, *Adgre5*^+/+^ Ub-GFP^+^ or *Adgre5*^−/−^ Ub-GFP^+^ splenocytes were transferred into WT mice. 10 minutes later, TNP-Ficoll was injected intravenously and GFP^+^ cells from blood were analyzed 40 minutes later for bound TNP-Ficoll. Representative histogram (left panel) and MFI (right panel) of TNP-Ficoll level on transferred *Adgre5*^−/−^ (n = 8) and *Adgre5*^+/+^ (n = 8) MZ B cells in blood. **e**, Frequencies of B1a and B1b cells in peritoneal cavity in *Adgre5*^−/−^ (n = 13) and control (n = 13) mice. **f**, Representative histogram (left panel) and MFI (right panel) of TNP-Ficoll level on B1a and B1b cells in peritoneal cavity of *Adgre5*^−/−^ (n = 8) and control (n = 8) mice 40 minutes after intravenous TNP-Ficoll immunization. (**g**) Frequencies of IgH^a^ WT (*Adgre5*^+/+^), IgH^b^ *Adgre5*^+/+^ (n = 12) or *Adgre5*^−/−^ (n = 12) in total splenocytes of mixed chimeras. Data are pooled from three (**a**, **e**, **g**) or two (**b**-**d**, **f**) independent experiments. Each symbol represents one mouse and lines denote means. Statistical significance was tested by two-tailed *t*-test (**a**, **b**, **d**, **f**), or two-way ANOVA followed by Sidak’s multiple comparisons test (**c**, **e**) or one-way ANOVA followed by Tukey’s multiple comparisons test (**g**). Source data

MZ B cells have an established role in mounting rapid antibody responses against circulating T cell-independent antigens, such as bacterial capsular polysaccharides^4^. Trinitrophenol (TNP)-haptenated Ficoll, a large highly branched polysaccharide, is widely used as a model T cell-independent antigen. Like other polysaccharide antigens, Ficoll becomes rapidly coated with complement fragments following systemic injection, and the complex can bind to MZ B cells via complement receptors^38,39^. Forty minutes after injection, the amount of TNP–Ficoll bound to MZ B cells was lower in *Cd97*-KO mice than in littermate control mice (Fig. 7a). This defect was cell intrinsic, as *Cd97*-KO MZ B cells bound less TNP–Ficoll than WT B cells in mixed BM chimeras (Fig. 7b). Similar findings were made in mice lacking Gα_13_ in B cells (Fig. 7c). CD55-deficient and ARHGEF1-deficient mice also showed reductions in TNP–Ficoll capture (Extended Data Fig. 7a–c). These data indicate that the MZ B cells remaining in CD97 pathway-deficient mice are altered in their ability to capture a blood-borne antigen. When spleen B cells were injected intravenously just before treating mice with TNP–Ficoll, WT and *Cd97*-KO MZ B cells in blood circulation captured Ficoll with similar efficiency (Extended Data Fig. 7d). B1 cells can also contribute to T cell-independent antibody responses^5^. However, peritoneal B1 cells showed minimal capture of intravenously injected TNP–Ficoll, and binding was unaffected by CD97 deficiency (Extended Data Fig. 7e,f). To test the impact of B cell CD97 deficiency on the antibody response, IgH^a^:IgH^b^ mixed BM chimeras were generated by combining *Cd97*-KO (or control) IgH^b^ BM with BM from WT mice congenic for the IgH^a^ locus. Flow cytometric analysis confirmed that, compared to their frequency in control mixed chimeras, *Cd97*-KO IgH^b^ MZ B cells were reduced in WT IgH^a^:*Cd97*-KO IgH^b^ mixed chimeras (Extended Data Fig. 7g). At day 6 after TNP–Ficoll immunization, serum was collected, and the amount of TNP binding IgM^a^ and IgM^b^ was measured. Although the production of TNP-specific IgM^a^ from WT cells was equivalent in each type of mixed BM chimera, the production of TNP-specific IgM^b^ by *Cd97*-KO cells was significantly reduced (Fig. 7d). Immunization of the same types of mixed chimeras with the T cell-dependent antigen NP-CGG led to IgM and IgG1 responses at day 12 that were unaffected by CD97 deficiency (Fig. 7e,f). Finally, we examined the IgM response to TNP–Ficoll in CD97-deficient mice and found that it was reduced (Fig. 7g). Thus, CD97 is required for mounting an intact early IgM response against a blood-borne polysaccharide antigen.

## Discussion

MZ B cells migrate extensively within the blood-exposed MZ, yet they are rarely lost into blood circulation. Here, we show that MZ B cells sense their exposure to blood flow through interactions with passing RBCs using the CD55–CD97 ligand–receptor system. In the absence of CD97, CD55 or downstream signaling proteins Gα_13_ and ARHGEF1, MZ B cell homeostasis in the spleen cannot be maintained due to cell loss into blood circulation. MZ B cell CD97 engagement by passing CD55^+^ RBCs leads to extraction of the inhibitory CD97 NTF and activation of the receptor. In vitro studies in transfected cells establish that CD97 signaling causes Rho activation and cell membrane retraction, and we speculate that this retraction response favors MZ B cell retention in the MZ rather than movement into blood flow. This guidance system is important in allowing the spleen to mount serum IgM responses to blood-borne T cell-independent antigens.

The adhesion GPCR family has 33 members^13,40^. Recent structural studies of six family members have provided support for the conclusion that activation of the GPCR domain occurs after binding of a tethered ligand that corresponds to the residues immediately following the GPS^41^. The structures indicate that exposure of the tethered ligand can occur by removal of the NTF or possibly by it undergoing a conformational change that frees the tethered ligand for access to the GPCR binding site. Our studies suggest that CD97 activation involves NTF extraction. However, because the exposure to RBCs in vitro or in vivo only causes a partial reduction in CD97 staining, we do not exclude the possibility that receptor activation may also occur by conformational change without NTF extraction.

An important question is whether the interaction strength between CD55 and CD97 is strong enough for NTF extraction to occur. A recent structural study found that the total buried surface for the human CD97–CD55 interaction was 1,920 Å (ref. ^2^), an interface area above the average for protein–protein interactions^42^. Moreover, the authors noted that the architecture of the CD55–CD97 binding interface leads to a shearing stretch geometry that is predicted to resist force applied to the protein interface. Affinity measurements have not been made under shear stress conditions, and thus information is not yet available for the relevant off rate of the interaction. It is possible that catch bonds (that is, bonds whose *k*_off_ decreases with increasing force) strengthen the interaction under shear stress conditions. Selectin–ligand binding strength is increased under appropriate shear stress conditions due to catch bonds^43^. Moreover, EGF domains can participate in catch bond formation^44^.

The retention of MZ B cells in the MZ depends on α_L_β_2_ and α_4_β_1_ integrins and the ligands ICAM1 and VCAM1 (ref. ^9^). Integrins are mechanosensitive receptors^45^, and their activity in MZ B cells may be promoted by the fluid shear stress experienced by the cells. We suggest that when MZ B cells move into regions of the MZ (or adjacent red pulp) with higher amounts of fluid flow, the encounter with RBCs and subsequent signaling via CD97 to Rho may cause an increase in integrin-mediated adhesion^46,47^. Increased adhesion may cooperate with Rho-mediated leading-edge retraction to favor cell retention and movement away from the region of high blood flow. The CD97 PBM contributed to membrane retraction in transfected HEK293T cells, in agreement with earlier data showing that it binds cytoskeletal proteins^21^. Although our tether force and RhoA activation measurements in HEK293T cells were consistent with the C-terminal motif having a functional role, it should be noted that these studies were performed with mScarlet fused to the CD97 C terminus, and this may have obstructed PDZ domain access. Further studies will be needed to define the role of this motif in MZ B cells.

CD97 pathway deficiency led to a reduction in the serum IgM response to a blood-borne T cell-independent antigen most likely due to the combined effects of reduced numbers of MZ B cells and the reduced ability of the remaining MZ B cells to encounter the antigen. We suggest that the reduced TNP–Ficoll encounter by the remaining cells is because they are more deeply lodged among other MZ cells and are less exposed to blood flow. Our data indicate that Gα_13_ and ARHGEF1 function downstream of CD97 to support behaviors needed for efficient blood-borne antigen capture. However, we note that these signaling molecules have roles downstream of additional receptors, and this likely explains the broader B cell response defects that have been observed in some studies of Gα_13_- and ARHGEF1-deficient mice^27,48,49^.

The human spleen has a more complex MZ architecture than the mouse spleen, and the cellular dynamics are less understood^1,3,32^. However, a marginal sinus has been identified, and at least some regions of the MZ are exposed to the open blood circulation^50^. Moreover, splenectomy in humans is associated with a reduced ability to control systemic infections by encapsulated bacteria^3^, and human MZ B cells are involved in responses against bacterially derived polysaccharides^32^. Our finding of high CD97 expression on human MZ B cells and sensitivity of the NTF to extraction by engagement with RBCs raise the possibility that the pathway we identified in mice may also be involved in human spleen function in responding to blood-borne pathogens. Future studies, for example, with CD97-blocking reagents in primates, will be needed to understand the importance of this pathway in humans.

## Methods

### Mice

B6 (NCI 556) and B6-Ly5.1 (CD45.1; NCI 564) mice were purchased from the National Institute at Charles River at age 6–8 weeks. *Mpl*^−/−^ (MGI, 3763248) mice were provided by M. R. Looney (University of California, San Francisco; UCSF). *Rag1*^−/−^ (JAX, 002216; B6.129S7-*Rag1*^*tm1Mom*^/J) mice were provided by A. Ma (UCSF). *Mb1-cre* mice (JAX, 020505; B6.C(Cg)-*Cd79a*^*tm1(cre)Reth*^/EhobJ), IgH^a^ congenic B6 mice (JAX, 001317; B6.Cg-*Gpi1*^*a*^ *Thy1*^*a*^ *Igh*^*a*^/J), *Adgre5*^−/−^ mice^17^, *Cd55*^−/−^ mice^17^, *Arhgef1*^−/−^ mice^51^, *Gna13*^fl/fl^ mice^52^, Ub-GFP mice (JAX, 004353; Tg(UBC-GFP)30Scha/J) and *Cd19*^−/−^ mice^53^ were from the internal colony. All mice were on a C57BL/6 background. All mice were housed in a specific pathogen-free environment at the Laboratory of Animal Research Center at UCSF, and all animal procedures were approved by the UCSF Institutional Animal Use and Care Committee. Mice of both sexes were used within an age range of 8–22 weeks. To block CD97, mice were treated with 30 μg of purified anti-CD97 (MAB33734, R&D) injected intravenously, and mice were analyzed 3 h or 4 d after treatment. For BM chimeras, mice were irradiated with 5.5 Gy of γ-irradiation in two doses 3 h apart and then intravenously transferred with BM cells from donors of the indicated genotypes. Chimeras were analyzed 8–12 weeks after reconstitution. In vivo pulse labeling was performed by injecting 1 μg of PE-conjugated anti-CD45.1 or anti-CD45.2 intravenously, and mice were analyzed after 3 min. Mice were allocated to control and experimental groups randomly. No statistical methods were used to predetermine sample sizes, but our sample sizes are similar to those reported in previous publications^8,48,54^. Data collection and analysis were not performed blind to the conditions of the experiments. No data points were excluded from the analyses for any reason. No animals were excluded except in rare cases where BM chimeras became sick before they could be used in an experiment.

### Retroviral constructs and BM transductions

The CD97 gene *Adgre5* encodes isoforms of CD97 with different numbers of N-terminal EGF domains, with ADGRE5 (1,2,x,3,4, where x is a short peptide) and ADGRE5 (1,2,4) being the prominent isoforms. CD55 interacts predominantly with CD97 EGF domains 1 and 2, and it binds to all of the isoforms^42,55^. By performing PCR on cDNA prepared from sorted WT MZ B cells, we found that ADGRE5 (1,2,4) was abundant (unpublished observations), and we therefore used this variant for our studies. The use of the shorter variant allowed for improved expression from retroviral constructs. ADGRE5 (1,2,4) and its mutants joined to Thy1.1 by a 2A self-cleaving peptide sequence or ADGRE5 (1,2,4) with a C-terminal GFP fusion were inserted into a pQEF Moloney murine leukemia virus retroviral vector that incorporates an *EF1* promoter for improved expression. The Plat-E cell line, a gift from S. R. Schwab (New York University), was used to package virus. To prepare for the BM transduction, donor mice were intravenously injected with 3 mg of 5-fluorouracil (Sigma). BM was collected after 4–5 d and cultured with complete DMEM supplemented with interleukin-3 (IL-3), IL-6 and stem cell factor. BM cells were spin infected at days 1 and 2 and were subsequently injected into irradiated recipients after the second infection.

### Immunizations

For the TNP–Ficoll uptake assay, 40 μg of TNP–Ficoll (Biosearch Technologies) was intravenously injected into each mouse 40 min before analysis. For analysis of the early antibody response, chimeras were immunized with 10 μg of TNP–Ficoll intravenously, and serum was collected 5–6 d after immunization. For analysis of T cell-dependent antibody responses, chimeras were treated with 50 μg of NP-CGG (Biosearch Technologies) in 50 μl of aluminum hydroxide gel by intraperitoneal injection, and serum was collected 12 d after immunization. TNP- or NP-specific antibodies were measured by ELISA using plates coated with TNP- or NP-BSA (Biosearch Technologies). TNP- or NP-specific antibodies were detected with biotinylated antibodies to mouse IgM^a^ (DS-1, 553515; 1:300), IgM^b^ (AF6-78, 553519; 1:300), IgG1^a^ (10.9, 553500; 1:300) and IgG1^b^ (B68-2, 553533, BD Biosciences; 1:300), followed by peroxidase-conjugated streptavidin (016-030-084, 1 mg ml^−1^, Jackson ImmunoResearch; 1:500). ELISA plates were developed using a Substrate Reagent Pack (R&D) and read on a VERSA MAX microplate reader at an optical density of 450 nm.

### Immunofluorescence staining

Spleen tissue cryosections (10 μm) were fixed in acetone for 10 min, dried for 1 h and subjected to staining with goat anti-IgD, AF647-conjugated anti-IgM (RMM-1, 406526; 1:100), APC-conjugated anti-IgM^a^ (MA-69, 408613; 1:100) and PE-conjugated anti-IgM^b^ (AF6-78, 406208; 1:100) from Biolegend and AMCA-conjugated donkey anti-goat IgG (H + L; 705-155-147, 0.5 mg ml^−1^; 1:200) from Jackson Immunoresearch. Images were captured using a Zeiss AxioOberver Z1 inverted microscope and stitched together using ZEN 2 (blue edition).

### Flow cytometry

Single-cell suspensions of splenic cells were prepared and stained with antibodies of indicated specificities in MACS buffer (PBS and 1% fetal bovine serum). The antibodies used for staining were BV785-conjugated anti-B220 (RA3-6B2, 103246; 1:200), BV605-conjugated anti-CD19 (6D5, 115540; 1:200), Pacific Blue-conjugated anti-CD21/CD35 (7E9, 123414; 1:200), PE/Cy7-conjugated anti-CD23 (B3B4, 101614; 1:200), PE-conjugated anti-CD5 (53-7.3, 100608; 1:200), PerCP-Cy5.5-conjugated anti-IgM (RMM-1, 406512; 1:200), PE-conjugated anti-CD45.2 (104, 109808; 1:200), FITC-conjugated anti-CD45.1 (A20, 110706; 1:200), FITC-conjugated anti-CD1d (1B1, 123508; 1:200), APC-conjugated anti-TER-119 (TER-119, 116212; 1:200), FITC-conjugated anti-CD41 (MWReg30, 133903; 1:200), PE-conjugated anti-IgD (IA6-2, 348204; 1:200), Pacific Blue-conjugated anti-IgM (MHM-88, 314514; 1:200), AF700-conjugated anti-CD1c (L161, 331529; 1:200) and FITC-conjugated anti-CD27 (M-T271, 356404; 1:200) from Biolegend; APC hamster IgG1 isotype control (anti-TNP, A19-3, 553974; 1:200), AF647-conjugated anti-Ki-67 (B56, 558615; 1:200) and PE hamster IgG1 isotype control (anti-TNP, A19-3, 553972; 1:200) from BD Biosciences; PE-conjugated anti-CD55 (REA300, 130-104-023; 1:100), APC-conjugated anti-CD97 (REA678, 130-110-229; 1:100), Annexin V-FITC (130-093-060; 1:200), APC-conjugated anti-CD97 (REA1242, 130-124-980; 1:100) and APC-conjugated anti-CD55 (REA1231, 130-124-497; 1:100) from Miltenyi Biotec. Dead cell exclusion was based on Fixable Viability Dye eFluor 780 staining (eBioscience), and non-singlet events were excluded with FSC-W/FSC-H characteristics. All data were collected on an LSRII and Symphony A1 cytometer (BD) with BD FACSDIVA V8.0.1 and 9.0.2 and analyzed with FlowJo V10 (TreeStar).

### RBC enrichment and transfusion

Blood (400 μl) was collected retro-orbitally from *CD55*^−/−^ or *CD55*^+/+^ mice into Alserver’s solution. The blood was mixed and centrifuged at 100*g* for 10 min at 24 °C without brake. Platelet-rich plasma and leukocyte-rich buffy coat were removed, and the remaining *CD55*^−/−^ or *CD55*^+/+^ RBCs were transferred intravenously into the appropriate mice once per week. Mice were analyzed 4 weeks after blood transfusion.

### Human samples and processing

Adult human splenic tissue was obtained from research-consented deceased organ donors at the time of organ acquisition for clinical transplantation through an institutional review board (IRB)-approved research protocol with Donor Network West, the organ procurement organization for Northern California, in collaboration with the UCSF Viable Tissue Acquisition Lab (VITAL) Core. The study and all VITAL core studies are UCSF IRB designated as non-human subjects research (UCSF Human Research Protection Program IRB, study 20-31618, reference 299695), as tissues are from deidentified deceased individuals. Splenic tissue was collected immediately after clinical organ procurement, stored and transported in University of Wisconsin preservation medium on ice and delivered at the same time as organs for transplantation for immediate processing. Donor spleens were cut into small pieces and homogenized and filtered through a 100-μm cell strainer. RBCs were lysed, and samples were prepared as single-cell suspensions. Human blood was collected into heparin-coated tubes, and RBCs were enriched by centrifugation at 100*g* for 10 min without brake.

### Shear flow model in vitro

To mimic shear flow in vitro, 100 μl of purified RBCs was introduced into 1.5-ml tubes containing splenocytes from mice or humans and shaken (Eppendorf ThermoMixer F1.5) at 24 °C at 1,000 r.p.m. (shear ~14 dyne cm^−2^) or not agitated. Forty-five minutes later, cells were collected, lysed and analyzed for CD97 expression by flow cytometry.

### Cell morphology in CD97-expressing HEK293T cells

HEK293T cells (originally from ATCC) were transfected with CD97 (WT)–GFP, CD97 (T419G)–GFP or CTF–GFP fusion constructs. Cell membranes (CellBrite Steady 550 Membrane Staining kit, Biotium), nucleus (Hoechst 33342, Thermo Fisher) and surface CD97 (APC-conjugated anti-CD97, Miltenyibiotec) were stained in live cells and imaged 40 h after transfection. Imaging was conducted with a STELLARIS 8 confocal microscope (Leica) with a water immersion ×20 objective lens at a *z* step of 1 μm. Images were processed with Imaris software (Bitplane). Longest diameters of transfected individual cells were measured with ImageJ.

### Lentiviral constructs and generation of CD97-expressing cell lines

ADGRE5 (1,2,4), its mutant T419G or PBM (that lacks the C-terminal SSESGM) with a C-terminal Scarlet fusion was inserted into the pLenti-SFFV Puro Lentiviral vector. Anillin–GFP RhoA biosensor was cloned from pEGFP-RhoA Biosensor (a gift from M. Glotzer, University of Chicago, Addgene plasmid 68026) and inserted into the lentiviral vector. Lentivirus were generated by mixing the plasmids HIV-gag pol (4 μg), vesicular stomatitis virus G protein (0.57 μg) and the respective lentiviral constructs (4 μg) and incubating this transfection mix in optimum medium (Thermo Fisher) with 4 μl of Xtremegene (Clontech) for 20 min at room temperature. Transfection solution was then applied to a 10-cm tissue culture-treated dish with seeded 4.4 × 10^6^ HEK293T cells. Supernatants were collected at 24 and 48 h after transfection, filtered through a 0.45-µm filter and supplemented with 20 mM HEPES (pH 7.4) and 20 µM polybrene before being aliquoted and frozen at −80 °C. HEK293T cells were sequentially transduced with the lentivirus by diluting 1:2 with complete medium (DMEM, 10% fetal bovine serum and 1% penicillin/streptomycin), centrifuged for 2 h at 935*g* at 37 °C, expanded and sorted.

### Optical C-trap plasma membrane tension measurements

Plasma membrane tension measurements were performed using a C-Trap optical trapping instrument (Lumicks BV). An IR laser beam (50 mW, 1,064 nm) was tightly focused through a series of mirrors, beam expanders and a high-numerical-aperture objective lens (×63/1.2-NA, water immersion, Nikon) to form a steerable optical trap. Cells were immobilized in an Ibidi μ-slide 0.4 Luer glass bottom (Ibidi) treated with 100 μl of 20 μg ml^−1^ fibronectin (Sigma). Mouse CD55 protein (R&D) was biotinylated with an EZ-Link Micro Sulfo-NHS-LC-Biotinylation kit (Thermo Fisher), according to the manufacturer’s instructions. To measure plasma membrane tension, polystyrene beads (2.2 μm, Spherotech) coated with 20 μg of biotinylated mouse CD55 were added to the cell culture medium inside the slide. The beads were briefly placed in contact with the cell membrane (30 s), and tethers were extruded by moving the bead away from the cell perpendicularly at a speed of 2 µm s^−1^. Force measurements were performed in the Lumicks Bluelake software suite by capturing the exiting trapping light with a high-numerical-aperture condenser lens (×63/1.45-NA, oil immersion, Zeiss) and measuring bead displacement in the trap with position-sensitive detectors through back focal plane interferometry. Florescence imaging was performed at 488 nm (RhoA anillin reporter) and 561 nm (CD97 WT or CD97 T419G expression) λ as membrane tethers were being pulled to ascertain RhoA activation at the site of bead contact and membrane tether. For RhoA recruitment/activation analysis, a region of interest was drawn around the site of bead attachment and pulling. The intensity of the anillin–GFP RhoA biosensor was measured over time in the 488-nm florescence channel. The region of interest size was maintained for all images analyzed. Images were analyzed using Fiji and rendered as videos with Adobe AfterEffect. All videos are played back at 5 frames per s, and time stamps are presented in minutes:seconds.

### Intravital imaging of MZ B cells in spleen

MZ B cells were imaged in *Cd19*^−/−^ mice reconstituted with GFP B cells. CTV-labeled naive B cells (4 × 10^7^) were adoptively transferred intravenously 1 or 2 d before imaging. PKH26 dye-labeled RBCs (50 μl) were intravenously injected 3 h before imaging. Texas Red dextran (70 kD) was intravenously injected 30 min before imaging. The basic setup and procedure for intravital two-photon imaging of mouse spleens were essentially the same as previously described^8^. Imaging was conducted with STELLARIS 8 (Leica) using a two-photon microscope equipped with two Chameleon lasers at a *z* step of 3 μm. The imaging depth under the capsule of the spleen was between ~ 50 μm and ~200 μm. For video acquisition, a series of images was collected every 20–35 s. Excitation wavelengths were 820 nm and 1,030 nm. Off-line analyses were conducted with Imaris V9.3.1 (Bitplane). Cell count was analyzed with Imaris automatic spot tracking aided by manual verification, and cell migration and interaction were analyzed and verified with manual tracking in three-dimensional views. To distinguish PKH26^+^ RBCs from macrophages that had engulfed damaged PKH26^+^ RBCs, we took advantage of the observation that the macrophages also phagocytosed circulating Texas Red dextran and were thus fluorescent in both the red and far-red channels and applied a filter to remove bright far-red fluorescent cells from red fluorescent cells. Image sequences were rendered as videos with Adobe AfterEffect. All videos are played back at 20 frames per s unless indicated otherwise, and time stamps are represented in minutes:seconds.

### Statistical analysis

Statistics and graphing were performed with Prism 9.4.1 (GraphPad). Two-tailed Student’s *t*-tests were used to compare endpoint means of different groups. In grouped analyses, ordinary two-way ANOVAs were performed, and the indicated *P* values are from individual *t*-tests with Sidak’s multiple testing correction. When multiple comparisons were being performed, ordinary one-way ANOVAs with Tukey’s multiple-comparisons tests were used. Data distribution was assumed to be normal, but this was not formally tested. Single-cell sequencing data were analyzed with the Seurat R package.

### Reporting summary

Further information on research design is available in the Nature Portfolio Reporting Summary linked to this article.

## Online content

Any methods, additional references, Nature Portfolio reporting summaries, source data, extended data, supplementary information, acknowledgements, peer review information; details of author contributions and competing interests; and statements of data and code availability are available at 10.1038/s41590-023-01690-z.

### Supplementary information

Reporting Summary

Supplementary Video 1 A *z*-projection view illustrating reconstituted GFP^+^ MZ B cell distribution in *Cd19*^−/−^ mouse spleen. GFP^+^ B cells (green), PKH26-labeled RBCs (red) and CTV-labeled naive B cells (blue) were visualized in a *z*-projection view; 141-μm-thick *z* stack. The very bright GFP^+^ cells that are not adjacent to CTV^+^ naive B cells are most likely plasma cells. MZ B cells are most clearly viewed at the 8-s mark.

Supplementary Video 2 Examples of contacts between reconstituted GFP^+^ MZ B cells and RBCs in *Cd19*^−/−^ mice. GFP^+^ B cells (green), PKH26-labeled RBCs (red) and CTV-labeled naive B cells (blue) were visualized in 36-min time-lapse videos. The video is replayed with an enlarged view shown. Blue arrowheads highlight contacts between MZ B cells and RBCs; 21-μm-thick *z* stack; scale bar, 40 μm. See also corresponding Fig. 1e.

Supplementary Video 3 Examples of contacts between reconstituted GFP^+^ MZ B cells and RBCs in *Cd19*^−/−^ mice with tracks of RBCs. GFP^+^ B cells (green), PKH26-labeled RBCs (red) and CTV-labeled naive B cells (blue) were visualized in 16-min time-lapse videos. The video is replayed with an enlarged view shown with some tracks of RBCs (yellow lines). Blue arrowheads highlight contact interfaces between MZ B cells and RBCs; 30-μm-thick *z* stack; scale bar, 50 μm.

Supplementary Video 4 Examples of contacts between reconstituted GFP^+^ MZ B cells and RBCs in *Cd19*^−/−^ mice with tracks of MZ B cells. GFP^+^ B cells (green), PKH26-labeled RBCs (red) and CTV-labeled naive B cells (blue) were visualized in 28-min time-lapse videos. The video is replayed with an enlarged view shown and some tracks of MZ B cells (yellow lines). Blue arrowheads highlight contact interfaces between MZ B cells and RBCs; 21-μm-thick *z* stack; scale bar, 50 μm.

Supplementary Video 5 Example of reconstituted *Adgre5*^+/+^ GFP^+^ B cell and transferred RBC behaviors in *Cd19*^−/−^ mouse spleen. *Adgre5*^+/+^ GFP^+^ B cells (green), PKH26-labeled RBCs (red) and CTV-labeled naive B cells (blue) were visualized within the red pulp in an 18-min time-lapse video; 33-μm-thick *z* stack. The GFP^+^ cells in this region are likely a mixture of MZ B cells and plasma cells; scale bar, 50 μm. See also corresponding Fig. 2j.

Supplementary Video 6 Example of reconstituted *Adgre5*^−/−^ GFP^+^ B cell and transferred RBC behaviors in *Cd19*^−/−^ mouse spleen. *Adgre5*^−/−^ GFP^+^ B cells (green), PKH26-labeled RBCs (red) and CTV-labeled naive B cells (blue) were visualized within the red pulp in a 33-min time-lapse video. Blue arrowheads highlight cells entering a large sinus. Within sinuses, cells sometimes continued to migrate before being caught in flow and quickly moving out of view; 42-μm-thick *z* stack. The GFP^+^ cells in this region are likely a mixture of MZ B cells and plasma cells; scale bar, 50 μm. See also corresponding Fig. 2j.

Supplementary Video 7 First example of RhoA activation in WT CD97-expressing cells at the site of pulling of CD55-coated beads. HEK293T cells expressing WT CD97–Scarlet fusion protein (green) and active RhoA reporter (anillin–GFP; blue) were visualized in 38-s time-lapse videos taken on a C-trap. Each cell was preassociated with a trapped CD55-coated bead. The videos are played with a white circle highlighting the pulling area and a blue arrowhead highlighting the pulling point. The appearance of the blue arrowhead indicates the start of pulling of the bead, and the disappearance of the arrowhead indicates the end of pulling. Bead pulling began approximately 30 s after the bead contacted the cell. Background RhoA reporter activity was detected in other regions of the cell; scale bar, 5 μm. See also corresponding Fig. 6c–e.

Supplementary Video 8 Second example of RhoA activation in WT CD97-expressing cells at the site of pulling of CD55-coated beads. HEK293T cells expressing WT CD97–Scarlet fusion protein (green) and active RhoA reporter (anillin–GFP; blue) were visualized in 27-s time-lapse videos taken on a C-trap. Each cell was preassociated with a trapped CD55-coated bead. The videos are played with a white circle highlighting the pulling area and a blue arrowhead highlighting the pulling point. The appearance of the blue arrowhead indicates the start of pulling of the bead, and the disappearance of the arrowhead indicates the end of pulling. Bead pulling began approximately 30 s after the bead contacted the cell. Background RhoA reporter activity was detected in other regions of the cell; scale bar, 5 μm. See also corresponding Fig. 6c,d.

Supplementary Video 9 First example showing lack of RhoA activation in cells expressing CD97 T419G at the site of pulling of CD55-coated beads. HEK293T cells expressing CD97 T419G–Scarlet fusion protein (green) and active RhoA reporter (anillin–GFP; blue) were visualized in 30-s time-lapse videos taken on a C-trap. Each cell was preassociated with a trapped CD55-coated bead. The videos are played with a white circle highlighting the pulling area and a blue arrowhead highlighting the pulling point. The appearance of the blue arrowhead indicates the start of pulling of the bead, and the disappearance of the arrowhead indicates the end of pulling. Bead pulling began approximately 30 s after the bead contacted the cell. Background RhoA reporter activity was detected in other regions of the cell; scale bar, 5 μm. See also corresponding Fig. 6c,d,f.

Supplementary Video 10 Second example showing lack of RhoA activation in cells expressing CD97 T419G at the site of pulling of CD55-coated beads. HEK293T cells expressing CD97 T419G–Scarlet fusion protein (green) and active RhoA reporter (anillin–GFP; blue) were visualized in 22-s time-lapse videos taken on a C-trap. Each cell was preassociated with a trapped CD55-coated bead. The videos are played with a white circle highlighting the pulling area and a blue arrowhead highlighting the pulling point. The appearance of the blue arrowhead indicates the start of pulling of the bead, and the disappearance of the arrowhead indicates the end of pulling. Bead pulling began approximately 30 s after the bead contacted the cell. Background RhoA reporter activity was detected in other regions of the cell; scale bar, 5 μm. See also corresponding Fig. 6c,d.

Supplementary Video 11 First example showing extent of RhoA activation in CD97 PBM-expressing cells at the site of pulling of CD55-coated beads. HEK293T cells expressing CD97 PBM–Scarlet fusion protein and RhoA reporter (anillin–GFP; blue) were visualized in 15-s time-lapse videos taken on a C-trap. Each cell was preassociated with a trapped CD55-coated bead. The videos are played with a white circle highlighting the pulling area and a blue arrowhead highlighting the pulling point. The appearance of the blue arrowhead indicates the start of pulling of the bead, and the disappearance of the arrowhead indicates the end of pulling. Bead pulling began approximately 30 s after the bead contacted the cell. Background RhoA reporter activity was detected in other regions of the cell; scale bar, 3 μm. See also corresponding Fig. 6c,d,g.

Supplementary Video 12 Second example showing extent of RhoA activation in CD97 PBM-expressing cells at the site of pulling of CD55-coated beads. HEK293T cells expressing CD97 PBM–Scarlet fusion protein and RhoA reporter (anillin–GFP; blue) were visualized in 16-s time-lapse videos taken on a C-trap. Each cell was preassociated with a trapped CD55-coated bead. The videos are played with a white circle highlighting the pulling area and a blue arrowhead highlighting the pulling point. The appearance of the blue arrowhead indicates the start of pulling of the bead, and the disappearance of the arrowhead indicates the end of pulling. Bead pulling began approximately 30 s after the bead contacted the cell. Background RhoA reporter activity was detected in other regions of the cell; scale bar, 3 μm. See also corresponding Fig. 6c,d.

### Source data

Source Data Fig. 2 Statistical source data.

Source Data Fig. 3 Statistical source data.

Source Data Fig. 4 Statistical source data.

Source Data Fig. 5 Statistical source data.

Source Data Fig. 6 Statistical source data.

Source Data Fig. 7 Statistical source data.

Source Data Extended Data Fig. 1 Statistical source data.

Source Data Extended Data Fig. 2 Statistical source data.

Source Data Extended Data Fig. 3 Statistical source data.

Source Data Extended Data Fig. 4 Statistical source data.

Source Data Extended Data Fig. 5 Statistical source data.

Source Data Extended Data Fig. 7 Statistical source data.

## Data Availability

All data are available in the main text or the supplementary materials.
