# Supplementary material for: Dynamic encounters with red blood cells trigger splenic marginal zone B cell retention and function
Source: Nat Immunol. 2023 Dec 4;25(1):142–54. doi: 10.1038/s41590-023-01690-z (PMC10761324; doi:10.1038/s41590-023-01690-z)
Supplement: Supplementary file 1 — Reporting Summary [file 41590_2023_1690_MOESM1_ESM.pdf]

nature portfolio

Corresponding author(s): Jason G. Cyster; Dan Liu

Last updated by author(s): Oct 14, 2023

Reporting Summary

Nature Portfolio wishes to improve the reproducibility of the work that we publish. This form provides structure for consistency and transparency in reporting. For further information on Nature Portfolio policies, see our Editorial Policies and the Editorial Policy Checklist.

Statistics

For all statistical analyses, confirm that the following items are present in the figure legend, table legend, main text, or Methods section.

n/a	Confirmed	
☐	☒	The exact sample size (*n*) for each experimental group/condition, given as a discrete number and unit of measurement
☐	☒	A statement on whether measurements were taken from distinct samples or whether the same sample was measured repeatedly
☐	☒	The statistical test(s) used AND whether they are one- or two-sided<br>*Only common tests should be described solely by name; describe more complex techniques in the Methods section.*
☐	☒	A description of all covariates tested
☐	☒	A description of any assumptions or corrections, such as tests of normality and adjustment for multiple comparisons
☐	☒	A full description of the statistical parameters including central tendency (e.g. means) or other basic estimates (e.g. regression coefficient) AND variation (e.g. standard deviation) or associated estimates of uncertainty (e.g. confidence intervals)
☐	☒	For null hypothesis testing, the test statistic (e.g. *F*, *t*, *r*) with confidence intervals, effect sizes, degrees of freedom and *P* value noted<br>*Give P values as exact values whenever suitable.*
☒	☐	For Bayesian analysis, information on the choice of priors and Markov chain Monte Carlo settings
☒	☐	For hierarchical and complex designs, identification of the appropriate level for tests and full reporting of outcomes
☒	☐	Estimates of effect sizes (e.g. Cohen's *d*, Pearson's *r*), indicating how they were calculated

Our web collection on statistics for biologists contains articles on many of the points above.

Software and code

Policy information about availability of computer code

Data collection	Flow cytometry data were collected using BD FACSDIVA V8.0.1 and 9.0.2.<br>IF data were collected using ZEN 2 (blue edition).<br>Two photon imaging data were collected using STELLARIS 8.
Data analysis	Flowcytometry data were processed and analyzed using FlowJo V10.<br>Imaging data were processed and analyzed using Imaris V9.3.1.<br>Single cell sequencing data were analyzed using the Seurat R package.<br>Statistics and graphing were done with Prism 9.4.1 (GraphPad).

For manuscripts utilizing custom algorithms or software that are central to the research but not yet described in published literature, software must be made available to editors and reviewers. We strongly encourage code deposition in a community repository (e.g. GitHub). See the Nature Portfolio guidelines for submitting code & software for further information.

Data

Policy information about availability of data

All manuscripts must include a data availability statement. This statement should provide the following information, where applicable:

- Accession codes, unique identifiers, or web links for publicly available datasets
- A description of any restrictions on data availability
- For clinical datasets or third party data, please ensure that the statement adheres to our policy

All data are available in the main text or the supplementary materials.

Research involving human participants, their data, or biological material

Policy information about studies with human participants or human data. See also policy information about sex, gender (identity/presentation), and sexual orientation and race, ethnicity and racism.

Reporting on sex and gender	We have two donors, one male and one female.
Reporting on race, ethnicity, or other socially relevant groupings	The male donor is Hispanic/Latino and the female donor is White.
Population characteristics	The male donor was 45 years old with gall bladder symptoms.<br>The female donor was 53 years old. She saw s physician for Thyroid issues, and took vitamins and supplements.
Recruitment	Death from natural causes
Ethics oversight	Human splenic tissue was obtained from research-consented deceased organ donors at the time of organ acquisition for clinical transplantation through an IRB-approved research protocol with Donor Network West, the organ procurement organization for Northern California, in collaboration with the UCSF VIable Tissue Acquisition Lab (VITAL) Core. The study and all VITAL core studies are UCSF IRB-designated as non-human subjects research (UCSF Human Research Protection Program Institutional Review Board, study #20-31618, reference # 299695), as tissues are from de-identified deceased individuals.

Note that full information on the approval of the study protocol must also be provided in the manuscript.

Field-specific reporting

Please select the one below that is the best fit for your research. If you are not sure, read the appropriate sections before making your selection.

☒ Life sciences ☐ Behavioural & social sciences ☐ Ecological, evolutionary & environmental sciences

For a reference copy of the document with all sections, see nature.com/documents/nr-reporting-summary-flat.pdf

Life sciences study design

All studies must disclose on these points even when the disclosure is negative.

Sample size	Each sample size is indicated in the figure legend. Based on common experiences in the relevant disciplines, the sample size was set empirically to provide a reasonable statistical power of detecting biological effects. No statistical methods were used to pre-determine the sample size.
Data exclusions	No data were excluded from analyses.
Replication	All experimental findings were reproducible at least three independent experiments, as indicated in figure legends, unless explicitly indicated otherwise.
Randomization	Co-housed animals of indicated genotypes were randomly assigned to groups for comparison where applicable.<br>Because of the relative immaturity of the marginal zone and its compartments, we chose adult humans with autoimmune diseases as donors.
Blinding	No blinding was involved in experiments, as there was no subjective measurement in these experiments.

Reporting for specific materials, systems and methods

We require information from authors about some types of materials, experimental systems and methods used in many studies. Here, indicate whether each material, system or method listed is relevant to your study. If you are not sure if a list item applies to your research, read the appropriate section before selecting a response.

Materials & experimental systems

n/a	Involved in the study
☐	☒ Antibodies
☐	☒ Eukaryotic cell lines
☒	☐ Palaeontology and archaeology
☐	☒ Animals and other organisms
☒	☐ Clinical data
☒	☐ Dual use research of concern
☒	☐ Plants

Methods

n/a	Involved in the study
☒	☐ ChIP-seq
☐	☒ Flow cytometry
☒	☐ MRI-based neuroimaging

Antibodies

Antibodies used

The antibodies used for staining were BV785-conjugated anti-B220 (RA3-6B2, Cat #103246, 1:200), BV605-conjugated anti-CD19 (6D5, Cat #115540, 1:200), Pacific Blue-conjugated anti-CD21/35 (7E9, Cat #123414, 1:200), PE/Cyanine7 conjugated anti-CD23 (B3B4, Cat #101614, 1:200), PE-conjugated anti-CD5 (53-7.3, Cat #100608, 1:200), PerCP-Cy5.5-conjugated anti-IgM (RMM-1, Cat #406512, 1:200), PE-conjugated anti-CD45.2 (104, Cat #109808, 1:200), FITC-conjugated anti-CD45.1 (A20, Cat #110706, 1:200), FITC-conjugated anti-CD1d (1B1, Cat #123508, 1:200), APC-conjugated anti-TER-119 (TER-119, Cat #116212, 1:200), FITC-conjugated anti-CD41 (MWReg30, Cat #133903, 1:200), PE-conjugated anti-IgD (IA6-2, Cat #348204, 1:200), Pacific Blue-conjugated anti-IgM (MHM-88, Cat #314514, 1:200), Alexa Fluor 700-conjugated anti-CD1c (L161, Cat #331529, 1:200), FITC-conjugated anti-CD27 (M-T271, Cat #356404, 1:200), Alexa Fluor 647-conjugated anti-IgM (RMM-1, Cat #406526, 1:100), APC-conjugated anti-IgMa (MA-69, Cat #408613, 1:100) and PE-conjugated anti-IgMb (AF6-78, Cat #406208, 1:100) from Biolegend; APC Hamster IgG1 isotype control (anti-TNP, A19-3, Cat #553974, 1:200), Alexa Fluor 647-conjugated anti-Ki-67 (B56, Cat #558615, 1:200), PE Hamster IgG1 isotype control (anti-TNP, A19-3, Cat #553972, 1:200), biotinylated anti-IgMa (DS-1, Cat #553515, 1:300), biotinylated anti-IgMb (AF6-78, Cat #553519, 1:300), biotinylated anti-IgG1a (10.9, Cat #553500, 1:300) and biotinylated anti-IgG1b (B68-2, Cat #553533, 1:300) from BD Biosciences; PE-conjugated anti-CD55 (REA300, Cat #130-104-023, 1:100), APC-conjugated anti-CD97 (REA678, Cat #130-110-229, 1:100), Annexin V-FITC (Cat #130-093-060, 1:200), APC-conjugated anti-CD97 (REA1242, Cat #130-124-980, 1:100) and APC-conjugated anti-CD55 (REA1231, Cat #130-124-497, 1:100) from Miltenyibiotec; AMCA-conjugated donkey anti-goat IgG (H+L) (Cat #705-155-147, 0.5 mg/ml, 1:200) and Peroxidase-conjugated Streptavidin (Cat #016-030-084,1 mg/ml, 1:500) from Jackson ImmunoResearch.

Validation

The specificity and application of these antibodies have been validated by the manufacturers on the website.
BV785-conjugated anti-B220
https://www.biolegend.com/fr-ch/products/brilliant-violet-785-anti-mouse-human-cd45r-b220-antibody-7960?GroupID=GROUP658
BV605-conjugated anti-CD19
https://www.biolegend.com/fr-fr/products/brilliant-violet-605-anti-mouse-cd19-antibody-7645?GroupID=BLG10556
Pacific Blue-conjugated anti-CD21/35
https://www.biolegend.com/en-us/cell-health/pacific-blue-anti-mouse-cd21-cd35-cr2-cr1-antibody-4336?GroupID=BLG5432
PE/Cyanine7 conjugated anti-CD23
https://www.biolegend.com/en-us/products/pe-cyanine7-anti-mouse-cd23-antibody-3941
PE-conjugated anti-CD5
https://www.biolegend.com/en-us/products/pe-anti-mouse-cd5-antibody-160?GroupID=BLG6762
PerCP-Cy5.5-conjugated anti-IgM
https://www.biolegend.com/en-us/products/percp-cyanine5-5-anti-mouse-igm-4519?GroupID=BLG3548
PE-conjugated anti-CD45.2
https://www.biolegend.com/en-us/cell-health/pe-anti-mouse-cd45-2-antibody-7?GroupID=BLG7007
FITC-conjugated anti-CD45.1
https://www.biolegend.com/en-us/products/fitc-anti-mouse-cd45-1-antibody-198?GroupID=BLG1933
FITC-conjugated anti-CD1d
https://www.biolegend.com/en-us/products/fitc-anti-mouse-cd1d-cd1-1-ly-38-antibody-4319?GroupID=BLG5435
APC-conjugated anti-TER-119
https://www.biolegend.com/en-us/products/apc-anti-mouse-ter-119-erythroid-cells-antibody-1863?GroupID=ImportedGROUP1
FITC-conjugated anti-CD41
https://www.biolegend.com/en-us/products/fitc-anti-mouse-cd41-antibody-5896
PE-conjugated anti-IgD
https://www.biolegend.com/en-us/products/pe-anti-mouse-igd-1379
Pacific Blue-conjugated anti-IgM
https://www.biolegend.com/en-us/search-results/pacific-blue-anti-human-igm-antibody-6637?GroupID=BLG4120
Alexa Fluor 700-conjugated anti-CD1c
https://www.biolegend.com/en-us/products/alexa-fluor-700-anti-human-cd1c-antibody-9864
FITC-conjugated anti-CD27
https://www.biolegend.com/en-us/products/fitc-anti-human-cd27-antibody-8348
Alexa Fluor 647-conjugated anti-IgM
https://www.biolegend.com/en-us/productstab/alexa-fluor-647-anti-mouse-igm-9684?GroupID=BLG3548
APC-conjugated anti-IgMa
https://www.biolegend.com/en-us/products/apc-anti-mouse-igma-antibody-15550
PE-conjugated anti-IgMb
https://www.biolegend.com/en-us/products/pe-anti-mouse-igmb-1745?GroupID=BLG4488
APC Hamster IgG1 isotype control
https://www.bdbiosciences.com/en-us/products/reagents/flow-cytometry-reagents/research-reagents/flow-cytometry-controls-and-lysates/apc-hamster-igg1-isotype-control.553974

Alexa Fluor 647-conjugated anti-Ki-67
https://www.bdbiosciences.com/en-us/products/reagents/flow-cytometry-reagents/research-reagents/single-color-antibodies-ruo/alexa-fluor-647-mouse-anti-ki-67.558615
PE Hamster IgG1 isotype control
https://www.bdbiosciences.com/en-us/products/reagents/flow-cytometry-reagents/research-reagents/flow-cytometry-controls-and-lysates/pe-hamster-igg1-isotype-control.553972
biotinylated anti-IgMa
https://www.bdbiosciences.com/en-us/products/reagents/flow-cytometry-reagents/research-reagents/single-color-antibodies-ruo/biotin-mouse-anti-mouse-igm-a.553515
biotinylated anti-IgMb
https://www.bdbiosciences.com/en-us/products/reagents/flow-cytometry-reagents/research-reagents/single-color-antibodies-ruo/biotin-mouse-anti-mouse-igm-b.553519
biotinylated anti-IgG1a
https://www.bdbiosciences.com/en-us/products/reagents/flow-cytometry-reagents/research-reagents/single-color-antibodies-ruo/biotin-mouse-anti-mouse-igg1-a.553500
biotinylated anti-IgG1b
https://www.bdbiosciences.com/en-us/products/reagents/flow-cytometry-reagents/research-reagents/single-color-antibodies-ruo/biotin-mouse-anti-mouse-igg1-b.553533
PE-conjugated anti-CD55
https://www.miltenyibiotec.com/US-en/products/cd55-daf-antibody-anti-mouse-reafinity-rea300.html#conjugate=pe:size=30-ug-in-1-ml
APC-conjugated anti-CD97
https://www.miltenyibiotec.com/US-en/products/cd97v2-antibody-anti-mouse-reafinity-rea678.html#conjugate=apc:size=30-ug-in-1-ml
Annexin V-FITC
https://www.miltenyibiotec.com/US-en/products/annexin-v-conjugates.html#conjugate=fitc:size=100-tests-in-1-ml
APC-conjugated anti-CD97
https://www.miltenyibiotec.com/US-en/products/cd97-antibody-anti-human-reafinity-rea1242.html#conjugate=apc:size=30-tests-in-60-ul
APC-conjugated anti-CD55
https://www.miltenyibiotec.com/US-en/products/cd55-daf-antibody-anti-human-reafinity-rea1231.html#conjugate=apc:size=30-tests-in-60-ul
AMCA-conjugated donkey anti-goat IgG (H+L)
https://www.jacksonimmuno.com/catalog/products/705-155-147
Peroxidase-conjugated Streptavidin
https://www.jacksonimmuno.com/catalog/products/016-030-084

Eukaryotic cell lines

Policy information about cell lines and Sex and Gender in Research

Cell line source(s)
The Plat-E cell line was a gift from Susan R. Schwab at New York University. HEK293T cell line was originally from ATCC.

Authentication
No specific procedure was taken to authenticate the cell line identity. The Plat-E cell line is selected in puromycin/blasticidin regularly.

Mycoplasma contamination
The cell lines have been tested negative for mycoplasma contamination.

Commonly misidentified lines (See ICLAC register)
No cell line used in this study are in the database of commonly misidentified cell lines.

Animals and other research organisms

Policy information about studies involving animals; ARRIVE guidelines recommended for reporting animal research, and Sex and Gender in Research

Laboratory animals
B6 (NCI 556) and B6-Ly5.2 (CD45.1) (NCI 564) mice were purchased from the National Institute at Charles River at age 6-8 weeks. Mpl −/− (MGI: 3763248) mice were provided by M. R. Looney, University of California, San Francisco (UCSF). Rag1−/− (JAX: 002216; B6.129S7-Rag1tm1Mom/J) mice were provided by A. Ma, UCSF. Mb1-cre mice (JAX: 020505; B6.C(Cg)-Cd79atm1(cre)Reth/EhobJ), IgHa congenic B6 mice (Jax: 001317; B6.Cg-Gpi1a Thy1a Igha/J), Adgre5−/− mice, Cd55−/− mice, Arhgef1−/− mice, Gna13f/f mice, Ubiquitin-GFP mice (JAX: 004353; Tg(UBC-GFP)30Scha/J), and Cd19−/− mice were from the internal colony. All mice were on a C57BL/6 background. All mice were housed in a specific-pathogen free environment at the Laboratory of Animal Research Center at UCSF and all animal procedures were approved by the UCSF Institutional Animal Use and Care Committee. In LARC, time controlled lightling on standard 12:12 light: dark cycle was applied. The humidity and ambient room temperature were maintained at 30% to 40% and 68ºF to 70ºF.

Ages (at the time of analysis) of the animals are listed below:
Fig. 1
Two-photon imaging was performed on two batches of MZ B cells reconstituted CD19-/- mice, one batch of mice were 18 weeks old, one batch of mice were 16 weeks old.

Fig. 2
1) In Fig. 2d, Adgre5-/- and Adgre5+/- mice were littermates and 11-12 weeks old.
2) In Fig. 2f and g, WT/Adgre5+/+ and WT/Adgre5-/- mixed chimeras were about 17-18 weeks old.
3) In Fig. 2h and i, B6 mice were 9-10 weeks old.
4) In Fig. 2k and i, BM chimeras were 14-15 weeks old.
Fig. 3
1) In Fig. 3b, G13f/f Mb1 cre+ (labeled as Gna13 cKO) mice and their littermate control mice were 10-12 weeks old.
2) In Fig. 3d, WT/Gna13 WT and WT/Gna13 cKO mixed chimeras were about 17-18 weeks old.
3) In Fig. 3e, G13f/f Mb1 cre+ (labeled as Gna13 cKO) mice and their littermate control mice were 8-9 weeks old.
4) In Fig. 3g, Arhgef1+/- and Arhgef1-/- mice were 12 weeks old.
5) In Fig. 3i, WT/Arhgef1+/+ and WT/Arhgef1-/- mixed chimeras were 16-17 weeks old.
Fig. 4
1) In Fig. 4c, CD55+/- and CD55-/- mice were 10-12 weeks old.
2) In Fig. 4e, WT/CD55+/+ and WT/CD55-/- mixed chimeras were about 15-16 weeks old.
3) In Fig. 4f, WT/CD55+/+, WT/dKO and CD55-/-/dKO mixed chimeras were about 17-18 weeks old.
4) In Fig. 4g, CD55+/- and CD55-/- mice were 15-16 weeks old.
5) In Fig. 4h, Mpl-/-/CD55+/- and Mpl-/-/CD55-/- mixed chimeras were 14 weeks old.
Fig. 5
1) In Fig. 5a, CD55+/- and CD55-/- mice were 15-16 weeks old.
2) In Fig. 5d, CD55+/+ and CD55-/- mice were 8 weeks old.
Fig. 7
1) In Fig. 7a, Adgre5-/- and Adgre5+/- mice were littermates and 12-14 weeks old.
2) In Fig. 7b, WT/Adgre5+/+ and WT/Adgre5-/- mixed chimeras were about 17-18 weeks old.
3) In Fig. 7c, Gna13 cKO mice and their littermate control mice were 10-12 weeks old.
4) In Fig. 7d, IgHa WT/IgHb Adgre5+/+ and IgHa WT/IgHb Adgre5-/- mixed chimeras were about 18-19 weeks old.
5) In Fig. 7e and f, IgHa WT/IgHb Adgre5+/+ and IgHa WT/IgHb Adgre5-/- mixed chimeras were about 20-22 weeks old.
6) In Fig. 7g, Adgre5-/- and Adgre5+/- mice were littermates and 13-14 weeks old.
Extended Data Fig. 2
In Extended Data Fig. 2g-j, Adgre5-/- GFP+, Adgre5+/+ GFP and B6 mice were 10-11 weeks old.
Extended Data Fig. 3
In Extended Data Fig. 3e, Gna12+/- and Gna12-/- chimeras were 17 weeks old.
Extended Data Fig. 4
In Extended Data Fig. 4i and j, Rag1-/-/CD55+/+ and Rag1-/-/CD55-/- mixed chimeras were 13 weeks old.
Extended Data Fig. 7
1) In Extended Data Fig. 7a, CD55-/- and littermate control mice were 11-12 weeks old.
2) In Extended Data Fig. 7b, Arhgef1-/- and littermate control mice were 11-12 weeks old.
3) In Extended Data Fig. 7c, WT/Arhgef1+/+ and WT/Arhgef1-/- mixed chimeras were 16 weeks old.

Wild animals	No wild animals was used in this study.
Reporting on sex	The study did not involve sex-biased study. Sex-matched mice were randomly chosen in each experiment.
Field-collected samples	The study did not involve samples collected from the field.
Ethics oversight	All animal procedures were approved by the UCSF Institutional Animal Use and Care Committee.

Note that full information on the approval of the study protocol must also be provided in the manuscript.

Flow Cytometry

Plots

Confirm that:

☒ The axis labels state the marker and fluorochrome used (e.g. CD4-FITC).

☒ The axis scales are clearly visible. Include numbers along axes only for bottom left plot of group (a 'group' is an analysis of identical markers).

☒ All plots are contour plots with outliers or pseudocolor plots.

☒ A numerical value for number of cells or percentage (with statistics) is provided.

Methodology

Sample preparation	Single-cell suspensions of splenic cells were prepared and stained with antibodies of indicated specificities in MACS buffer (PBS and 1% FBS).
Instrument	LSR II or Symphony A1 (BD Biosciences)
Software	Flowcytometry data were collected using BD FACSDIVA V8.0.1 and 9.0.2, and analyzed by FlowJo V10.
Cell population abundance	At least 10000 events were acquired in the defined cell population.

Gating strategy

Dead cell exclusion was based on Fixable Viability Dye eFluor 780 staining (eBioscience) and non-singlet events were excluded with FSC-W/FSC-H characteristics. Isotype control was used to discriminate bwtween background and marker-positive events.

☒ Tick this box to confirm that a figure exemplifying the gating strategy is provided in the Supplementary Information.
